# Variation in regulator of G-protein signaling 17 gene (*RGS17*) is associated with multiple substance dependence diagnoses

**DOI:** 10.1186/1744-9081-8-23

**Published:** 2012-05-16

**Authors:** Huiping Zhang, Fan Wang, Henry R Kranzler, Raymond F Anton, Joel Gelernter

**Affiliations:** 1 Departments of Psychiatry, Yale University School of Medicine, New Haven, CT, USA; 2 Departments of Genetics, Yale University School of Medicine, New Haven, CT, USA; 3 Departments of Neurobiology, Yale University School of Medicine, New Haven, CT, USA; 4 VA Connecticut Healthcare System, West Haven, CT, USA; 5 Department of Psychiatry, University of Pennsylvania Perelman School of Medicine and VISN4 MIRECC, Philadelphia VAMC, Philadelphia, PA, USA; 6 Department of Psychiatry and Behavioral Sciences, Medical University of South Carolina, Charleston, SC, USA

**Keywords:** *RGS17* and *RGS20*, Multiple substance dependence, Genetic association, Haplotype analysis, Regression analysis, Genotype-expression relationship

## Abstract

**Background:**

*RGS17* and *RGS20* encode two members of the regulator of G-protein signaling RGS-Rz subfamily. Variation in these genes may alter their transcription and thereby influence the function of G protein-coupled receptors, including opioid receptors, and modify risk for substance dependence.

**Methods:**

The association of 13 *RGS17* and eight *RGS20* tag single nucleotide polymorphisms (SNPs) was examined with four substance dependence diagnoses (alcohol (AD), cocaine (CD), opioid (OD) or marijuana (MjD)] in 1,905 African Americans (AAs: 1,562 cases and 343 controls) and 1,332 European Americans (EAs: 981 cases and 351 controls). Analyses were performed using both *χ*^2^ tests and logistic regression analyses that covaried sex, age, and ancestry proportion. Correlation of genotypes and mRNA expression levels was assessed by linear regression analyses.

**Results:**

Seven *RGS17* SNPs showed a significant association with at least one of the four dependence traits after a permutation-based correction for multiple testing (0.003≤*P*_*empirical*_≤0.037). The G allele of SNP rs596359, in the *RGS17* promoter region, was associated with AD, CD, OD, or MjD in both populations (0.005≤*P*_*empirical*_≤0.019). This allele was also associated with significantly lower mRNA expression levels of *RGS17* in YRI subjects (*P* = 0.002) and non-significantly lower mRNA expression levels of *RGS17* in CEU subjects (*P* = 0.185). No *RGS20* SNPs were associated with any of the four dependence traits in either population.

**Conclusions:**

This study demonstrated that variation in *RGS17* was associated with risk for substance dependence diagnoses in both AA and EA populations.

## Background

Substance (alcohol or drug) dependence (SD) is a set of complex disorders influenced by gene-gene and gene-environment interactions. Genes involved in dopaminergic, serotonergic, GABAergic, glutamatergic, cannabinoid, and opioidergic systems have been implicated in SD risk. Mounting evidence suggests that variation in genes coding for dopamine, serotonin, GABA, glutamate, cannabinoid, and opioid receptors may increase vulnerability to SD and related phenotypes [1-4]. The function of these receptors, which belong to a large G protein-coupled receptor (GPCR) family, is regulated by *regulators of G-protein signaling* proteins (RGSs) [5,6].

Members of the RGS family are functionally related to selective GPCR signal transduction pathways [7]. For example, they participate in opioid receptor desensitization, internalization, recycling and degradation [8,9]. In analyzing the function of RGS17 in mouse brain, Garzon *et al.*[10] found that, when RGS17 expression was reduced, the μ-opioid receptor (MOR)-mediated antinociceptive response to morphine and d-Ala^2^*N*-MePhe^4^, Gly-ol^5^-enkephalin (DAMGO) was increased. Ajit *et al.*[11] demonstrated that RGS17 interacted with protein kinase C interacting protein (PKCI-1) and modulated the signaling pathway of the MOR. Moreover, in membranes from periaqueductal gray matter (PAG), both RGS17 and RGS20 co-precipitated with the MOR [10]. By use of intraventricular administration of antisense oligonucleotides, Garzon *et al.*[12] demonstrated that a suppression of RGS20 expression in mouse brain greatly increased the supraspinal antinociceptive effect of the MOR agonists. Together, these findings indicate that GPCRs (e.g., MOR) are functionally linked to RGS proteins (e.g., RGS17 and RGS20).

RGS 17 and RGS20 are two members of the RGS-Rz subfamily. They are closely related to opioid receptors in both chromosomal location and cellular function. The RGS17 gene (*RGS17*) is linked to the μ-opioid receptor (MOR) gene (*OPRM1*) on chromosome 6 (distance: about 0.9 Mb), and the RGS20 gene (*RGS20*) is linked to the κ-opioid receptor (KOR) gene (*OPRK1*) on chromosome 8 (distance: about 0.6 Mb) [6]. Close genomic proximity may reflect a coordinated transcription of the linked genes or a shared regulatory mechanism for their expression [9]. In other words, the transcription of *RSG 17* (or *RGS20*) may influence the transcription of *OPRM1* (or *OPRK1*) and vice versa. Genomic proximity may also reflect a functional relationship between the RGS-Rz proteins (RGS17 and RGS20) and opioid receptors.

Several studies, including ours, have shown a positive association between variation at *OPRM1*[13-17] and *OPRK1*[18,19] and alcohol or drug dependence, although negative results have been reported [20]. Additionally, mouse genome scans have mapped a quantitative trait locus (QTL) for morphine preference to the μ-opioid receptor gene region (where *RGS17* is located) [21] and a QTL for alcohol consumption to the κ-opioid receptor gene region (where *RGS20* is located) [22]. Considering the close relationship between the RGS-Rz proteins and opioid receptors, and the association between opioid receptor genes and alcohol or drug dependence, we hypothesized that variation in *RGS17* and *RGS20* could affect vulnerability to various SD types. To date, no published studies have examined the association between *RGS17* and *RGS20* polymorphisms and SD or other psychiatric disorders, although associations between *RGS2* variants and anxiety [23,24] and between *RGS4* variants and schizophrenia [25,26] have been reported. We used a case–control association study approach to analyze the association of *RGS17* and *RGS20* variants and risk for four different SD diagnoses. We also examined the correlation between genotypes of SD-associated variants and gene expression levels.

## Methods

### Recruitment and ascertainment

Unrelated case and control subjects were recruited from substance abuse treatment centers and through advertisements at the University of Connecticut Health Center (n = 1,394), Yale University School of Medicine (APT foundation) (n = 1,256), the University of Pennsylvania School of Medicine (n = 304), and the Medical University of South Carolina (n = 283). Subjects gave informed consent as approved by the institutional review board at each clinical site, and certificates of confidentiality were obtained from the National Institute on Drug Abuse and the National Institute on Alcohol Abuse and Alcoholism. All subjects were interviewed using an electronic version of the Semi-Structured Assessment for Drug Dependence and Alcoholism (SSADDA) [27] to derive diagnoses for lifetime alcohol, cocaine, opioid or marijuana dependence (AD, CD, OD or MjD, respectively) according to DSM-IV criteria [28]. Control subjects were screened to exclude individuals with any of these four SD traits. Additionally, case and control subjects with a lifetime major psychotic disorder (schizophrenia or bipolar disorder) were excluded. The clinical characteristics (including SD comorbidity information) of participants are presented in Table 1. There were 1,562 unrelated AA cases: AD (n = 1,064, 68.1%), CD (n = 1,309, 83.8%), OD (n = 358, 22.9%) and/or MjD (n = 531, 34.0%) and 343 unrelated AA controls. There were 981 unrelated EA cases: AD (n = 671, 68.4%), CD (n = 696, 70.9%), OD (n = 577, 58.8%) and/or MjD (n = 318, 32.4%) and 351 EA unrelated controls.

**Table 1:** Clinical characteristics of case–control samples

	**African Americans (AAs)**		**European American (EAs)**	
	**SD cases (n = 1,562)**	**Contols (n = 343)**	**SD cases (n = 981)**	**Contols (n = 351)**
AD, n (%)	1064 (68.1%)	0 (0%)	671 (68.4%)	0 (0%)
CD, n (%)	1309 (83.8%)	0 (0%)	696 (70.9%)	0 (0%)
OD, n (%)	358 (22.9%)	0 (0%)	577 (58.8%)	0 (0%)
MjD, n (%)	531 (34.0%)	0 (0%)	318 (32.4%)	0 (0%)
AD + CD + OD + MjD, n(%)	87 (5.6%)	0 (0%)	131 (13.4%)	0 (0%)
AD + CD + OD, n(%)	196 (12.5%)	0 (0%)	283 (28.8%)	0 (0%)
AD + OD + MjD, n(%)	90 (5.8%)	0 (0%)	141 (14.4%)	0 (0%)
AD + CD + MjD, n(%)	360 (23.0%)	0 (0%)	195 (19.9%)	0 (0%)
CD + OD + MjD, n(%)	116 (7.4%)	0 (0%)	182 (18.6%)	0 (0%)
AD + CD, n(%)	854 (54.7%)	0 (0%)	476 (48.5%)	0 (0%)
AD + OD, n(%)	217 (13.9%)	0 (0%)	321 (32.7%)	0 (0%)
AD + MjD, n(%)	424 (27.1%)	0 (0%)	232 (23.6)	0 (0%)
CD + OD, n(%)	303 (19.4%)	0 (0%)	436 (44.4%)	0 (0%)
CD + MjD, n(%)	454 (29.1%)	0 (0%)	260 (26.5%)	0 (0%)
OD + MjD, n(%)	123 (7.9%)	0 (0%)	206 (21.0%)	0 (0%)
Male, n (%)	786 (50.5%)	153 (44.6%)	485 (49.4%)	190 (54.1%)
	χ^2^ = 3.45, df = 1, *P* = 0.063		χ^2^ = 1.70, df = 1, *P* = 0.192	
Age, years	39 ± 9	39 ± 9	38 ± 12	41 ± 13
	t = −1.029, *P* = 0.304 (2-tailed)		t = 4.76, *P* = 2.2 × 10^-6^ (2-tailed)	

*SD*, substance (alcohol, cocaine, opioid, and/or marijuana) dependence.

*AD*, alcohol dependence; *CD*, cocaine dependence; *OD*, opioid dependence; *MjD*, marijuana dependence.

Symbol " + " means comorbidity.

Sex differences between cases and controls were analyzed by the Chi-square test.

Age differences between cases and controls were analyzed by the *t*-test.

### Genotyping

Thirteen tag single nucleotide polymorphisms (SNPs) in *RGS17* and eight tag SNPs in *RGS20* were selected from public sources such as the NCBI dbSNP database (http://www.ncbi.nlm.nih.gov/SNP), the HapMap Genome Browser (http://www.hapmap.org), and the SNPbrowser software v 4.0 (Applied Biosystems), based upon their minor allele frequencies and linkage disequilibrium (LD) information (Table 2). The TaqMan method [29] was used to genotype SNP markers at the Yale University School of Medicine. Eight percent of genotypes were repeated for quality control; any mismatches triggered repeats of all genotypes on a given plate.

**Table 2:** **Characteristics of SNPs in*RGS20*and*RGS17***

**ID**	**SNPs**	**Chromosome**	**Gene**		**Allele**		
		**Position (hg18)**		**Location**		**MAF (AAs)**	**MAF (EAs)**
RGS17_1	rs9397578	153371201	*RGS17*	3' near gene	A/G	0.27 (A)	0.26 (A)
RGS17_2	rs7750874	153372161	*RGS17*	3' near gene	A/T	0.29 (A)	0.34 (A)
RGS17_3	rs503366	153375243	*RGS17*	Intron 4	C/T	0.47 (C)	0.49 (C)
RGS17_4	rs610614	153383477	*RGS17*	Intron 4	C/T	0.42 (C)	0.31 (C)
RGS17_5	rs545323	153387002	*RGS17*	Intron 4	C/T	0.05 (C)	0.33 (C)
RGS17_6	rs516557	153395551	*RGS17*	Intron 2	C/T	0.47 (T)	0.47 (C)
RGS17_7	rs9371276	153410854	*RGS17*	Intron 1	C/T	0.48 (T)	0.30 (C)
RGS17_8	rs1933258	153419500	*RGS17*	Intron 1	C/G	0.49 (G)	0.3 (C)
RGS17_9	rs9397585	153438568	*RGS17*	Intron 1	C/T	0.49 (C)	0.37 (C)
RGS17_10	rs685826	153452948	*RGS17*	Intron 1	C/T	0.46 (T)	0.44 (C)
RGS17_11	rs6931160	153472144	*RGS17*	Intron 1	C/G	0.50 (G)	0.44 (C)
RGS17_12	rs1281962	153473069	*RGS17*	Intron 1	C/G	0.24 (G)	0.46 (G)
RGS17_13	rs596359	153498746	*RGS17*	5' near gene	A/G	0.33 (G)	0.48 (G)
RGS20_1	rs1384797	54956481	*RGS20*	intron 1	A/G	0.40 (G)	0.02 (G)
RGS20_2	rs2220093	54963880	*RGS20*	intron 1	A/G	0.36 (A)	0.10 (G)
RGS20_3	rs1483537	54980301	*RGS20*	intron 1	A/G	0.35 (G)	0.01 (G)
RGS20_4	rs7824575	54984872	*RGS20*	intron 1	A/G	0.24 (G)	0.27 (A)
RGS20_5	rs2128821	55006166	*RGS20*	intron 1	C/G	0.42 (C)	0.26 (G)
RGS20_6	rs9298496	55018233	*RGS20*	intron 2	C/T	0.37 (C)	0.34 (C)
RGS20_7	rs6981243	55029044	*RGS20*	intron 3	A/C	0.42 (A)	0.41 (C)
RGS20_8	rs7009781	55035764	*RGS20*	downstream	C/T	0.27 (T)	0.17 (C)

Chromosome positions are based on Homo sapiens chromosome 6 genomic contig NT_025741.14 (*RGS17*) and chromosome 8 genomic contig NT_008183.18 (*RGS20*). MAF (AA), minor allele frequency in our African American (AA) sample; MAF (EA), minor allele frequency in our European Americans (EA) sample.

### *Statistical analysis*

Data analysis was conducted separately in AAs and EAs based on self-reported race. To verify the self-reported race, we used a Bayesian model-based clustering method implemented in the program STRUCTURE [30] to estimate the African and European ancestry proportions of individual subjects, using genotype data from 41 ancestry informative markers (AIMs), including 36 short tandem repeat markers and five SNPs, as described previously [31,32]. This clustering produced two distinct groups that were highly concordant with self-reported AA and EA group membership. Hardy-Weinberg equilibrium (HWE) analysis was carried out in control subjects for each of the 21 *RGS17* and the eight *RGS20* SNPs using the Chi-square test. Allelic association analyses were performed using the Pearson’s *χ*^2^ test. To adjust for the multiple tests performed and obtain an empirical null distribution of association test *P* values (*P*_empirical_), we conducted 10,000 permutations in the case–control sample. The association of SNP markers and SD traits was further evaluated using the multivariate logistic regression analysis under the additive model with consideration of possible confounding factors, which were sex, age, and ancestry proportion of subjects. The Cochran-Mantel-Haenszel (CMH) test was used to calculate the overall genetic effect of SNPs by combining data from AA and EA populations. The above four types of analyses were implemented using PLINK v.1.07 (http://pngu.mgh.harvard.edu/purcell/plink/) [33]. Haplotype analyses were carried out using the program Haploview v.4.2. [34]. Haplotype blocks were defined according to the criteria of Gabriel *et al.*[35].

### *Bioinformatics and genotype-expression analysis*

DNA sequences harboring SNP markers that showed a significant association with SD phenotypes were queried for predicted transcription factor (TF) binding sites using the computational tool of the Transcription Element Search System (TESS, http://www.cbil.upenn.edu/cgi-bin/tess). To assess the functional effect of SD-associated *RGS17* and *RGS20* variants on gene expression, whole genome Illumina lymphoblastoid cell line gene expression data from 120 unrelated HapMap individuals (60 from the CEU population and 60 from the YRI population) were extracted from the GSE6536 series data set in the Gene Expression Omnibus (GEO) site (http://www.ncbi.nlm.nih.gov/geo). Expression data (or mRNA levels) of *RGS17* (determined by probe GI_21361404-S), *RGS20* (determined by probe GI_13654234-A), and *OPRM1* (determined by probe GI_4505514-S) were included in the genotype-expression association analyses. Genotype data of *RGS17* and *RGS20* SNPs from 60 unrelated CEU individuals and 60 unrelated YRI individuals were downloaded from the HapMap genome browser (http://www.hapmap.org/cgi-perl/gbrowse/hapmap_B36). The correlation of SNP marker genotypes and mRNA expression levels was assessed by linear regression analyses assuming an additive model and adjusted by sex.

## Results

### *Allelic association*

**Figure 1:**
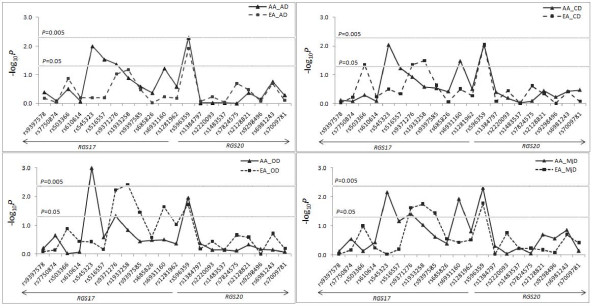
**Allelic association of 13*RGS17 *and eight *RGS2 *0 SNPs with four substance dependence traits.** X axis: *RGS17* and *RGS20* SNPs; Y axis: minus log_10_*P* values. AA: African Americans; EA: European Americans. AD: alcohol dependence; CD: cocaine dependence; OD: opioid dependence; and MjD: marijuana dependence.

There were no deviations from HWE for genotype distributions of any of the 13 *RGS17* and eight *RGS20* SNPs in either AA or EA controls (the *P* value for statistical significance was set at *P* > 0.05/21 = 0.002) (data not shown). As shown in Figure 1, Table 3 and Additional file 1: Table S1, seven *RGS17* SNPs showed significant association with at least one of the four SD traits after permutation-based correction for multiple testing (0.003≤*P*_*empirical*_≤0.037). Detailed information about genetic association results of 13 *RGS17* SNPs, *RGS17* physical position on Chromosome 6, and recombination rate in the gene region is presented in Additional file 1: Figures S1 and S2. SNP rs596359 (in the promoter region) was associated with AD, CD, OD and MjD in both AAs and EAs (0.005≤*P*_*empirical*_≤0.019). Six other SNPs (rs6931160 in Intron 1, rs9397585 in Intron 1, rs1933258 in Intron 1, rs9371276 in Intron 1, rs516557 in Intron 2 and rs545323 in Intron 4) were associated with one or more of these four SD traits in AAs and/or EAs (0.003≤*P*_*empirical*_≤0.037). Logistic regression analyses using sex, age and ancestry proportion as covariates confirmed the association of the seven *RGS17* SNPs with multiple SD traits in AAs and/or EAs (0.002≤ *P*_*adjusted*_≤0.053) (Table 3). Combining data from both AAs and EAs via meta-analysis showed that five *RGS17* SNPs (rs596359, rs6931160, rs1933258, rs9371276, and rs545323) were associated with at least one of the four SD traits (1.7 × 10^-4^≤*P*_*meta*_≤0.045). None of the eight *RGS20* SNPs was associated with any of the four SD traits in either AAs or EAs (Figure 1 and Additional file 1: Table S2).

**Table 3:** **Association of seven*RGS17*SNPs and four substance dependence (SD) Traits**

**SNPs**	**Trait**	**Race**	**RA**	**Frequency**	**χ**^**2**^	***P***_***obs***_	**OR (95%CI)**	***P***_***adj***_	**OR (95%CI)**	***P***_***emp***_
rs545323	AD	AA	C	0.06\0.03	6.70	0.010	1.96 (1.17−3.30)	0.013	1.96 (1.16−3.32)	0.010
	CD	AA	C	0.05\0.03	6.74	0.009	1.95 (1.17−3.25)	0.015	1.95 (1.16−3.28)	0.009
	OD	AA	C	0.07\0.03	11.27	0.001	2.55 (1.45−4.49)	0.002	2.51 (1.41−4.46)	0.003
	MjD	AA	C	0.06\0.03	7.30	0.007	2.10 (1.21−3.64)	0.009	2.10 (1.20−3.67)	0.009
rs516557	AD	AA	T	0.49\0.43	4.78	0.029	1.25 (1.02−1.52)	0.035	1.23 (1.02−1.50)	0.018
rs9371276	OD	EA	C	0.33\0.27	7.47	0.006	1.34 (1.09−1.66)	0.008	1.33 (1.08−1.63)	0.008
	MjD	EA	C	0.33\0.27	5.11	0.024	1.32 (1.04−1.62)	0.053	1.26 (1.00−1.58)	0.032
rs1933258	CD	EA	C	0.31\0.27	4.54	0.033	1.25 (1.02−1.54)	0.04	1.25 (1.01−1.54)	0.037
	OD	EA	C	0.33\0.27	8.51	0.004	1.37 (1.11−1.70)	0.004	1.36 (1.10−1.68)	0.005
	MjD	EA	C	0.33\0.27	5.63	0.018	1.34 (1.05−1.70)	0.036	1.29 (1.02−1.63)	0.024
rs9397585	OD	EA	C	0.40\0.35	4.39	0.036	1.24 (1.01−1.51)	0.02	1.27 (1.04−1.56)	0.028
	MjD	EA	C	0.41\0.35	4.36	0.037	1.27 (1.02−1.59)	0.035	1.28(1.02−1.61)	0.030
rs6931160	MjD	AA	G	0.47\0.53	6.35	0.012	0.78 (0.64−0.95)	0.015	0.79 (0.65−0.95)	0.016
	OD	EA	C	0.48\0.42	5.14	0.023	1.25 (1.03−1.51)	0.019	1.26 (1.04−1.54)	0.018
	CD	AA	G	0.49\0.53	4.56	0.033	0.83 (0.7−0.99)	0.036	0.83 (0.70−0.99)	0.028
rs596359	AD	AA	G	0.35\0.29	7.82	0.005	1.31 (1.08−1.58)	0.008	1.29 (1.07−1.56)	0.005
	AD	EA	G	0.50\0.44	6.37	0.012	1.27 (1.05−1.52)	0.011	1.27 (1.06−1.53)	0.014
	CD	AA	G	0.34\0.29	6.77	0.009	1.28 (1.06−1.54)	0.011	1.27 (1.06−1.53)	0.009
	CD	EA	G	0.51\0.44	6.91	0.009	1.28 (1.06−1.54)	0.008	1.29 (1.07−1.56)	0.006
	OD	AA	G	0.35\0.29	6.50	0.011	1.34 (1.07−1.69)	0.023	1.31 (1.04−1.63)	0.019
	OD	EA	G	0.5\0.44	5.54	0.019	1.26 (1.04−1.52)	0.01	1.29 (1.06−1.57)	0.013
	MjD	AA	G	0.35\0.29	8.05	0.005	1.35 (1.10−1.67)	0.008	1.33 (1.08−1.63)	0.006
	MjD	EA	G	0.51\0.44	5.70	0.017	1.30 (1.05−1.62)	0.015	1.31 (1.05−1.63)	0.013

*AA*, African Americans; *EA*, European Americans. *AD*, alcohol dependence; *CD*, cocaine dependence; *OD*, opioid dependence; *MjD*, Marijuana dependence.

*P*_obs_, observed *P* values using Pearson’s Chi-square tests; *P*_adj_, adjusted *P* values using multivariable logistic regression analyses after adjustment for sex, age and ancestry proportion under the additive model; *P*_emp_, empirical *P* values using permutation-based tests to correct for multiple testing.

RA, reference alleles; 95% CI, 95% confidence interval.

### *Haplotype association*

**Figure 2:**
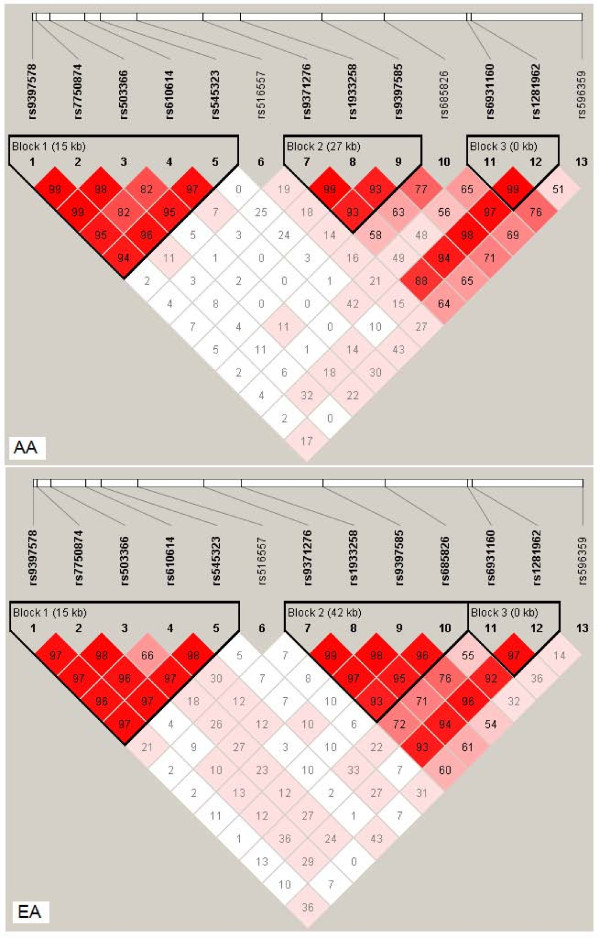
**Pairwise linkage disequilibrium (LD) between genotyped* RGS17 *SNPs.** Within each box is the pair-wise estimate of D’.

The association of *RGS17* variants with SD was further analyzed using the haplotype association analysis approach. As shown in Figure 2, *RGS17* SNPs were located in three haplotype blocks (I, II, and III) (Block II harbors three SNPs in AAs but four SNPs in EAs). Table 4 lists the haplotypes that were associated with one or more of the SD phenotypes (*P*_observed_ ≤ 0.05). In AAs, two haplotypes (GATTC and GTTCT) comprised of alleles of five *RGS17* SNPs (rs9397578-rs7750874-rs503366-rs610614-rs545323) (Block I in Figure 2) were associated with all four dependence traits (0.002 ≤ *P*_obs_ ≤ 0.024). The association between GATTC (potentially a risk haplotype) with OD and GTTCT (potentially a protective haplotype) with MjD remained significant after correction for multiple testing by permutation tests (GATTC with OD: *P*_empirical_ = 0.026; GTTCT with MjD: *P*_empirical_ =0.048). Two haplotypes (CCT and TGT) comprised of alleles of three *RGS17* SNPs (rs9371276-rs1933258-rs9397585) (Block II in Figure 2) and one haplotype (CC) comprised of alleles of two *RGS17* SNPs (rs6931160-rs1281962) (Block III in Figure 2) were only nominally associated with OD or MjD (0.021 ≤ *P*_obs_ ≤0.042). In EAs, three haplotypes, GTCTT comprised of alleles of five *RGS17* SNPs (rs9397578-rs7750874-rs503366-rs610614-rs545323) (Block I in Figure 2), CCCC comprised of alleles of four *RGS17* SNPs (rs9371276-rs1933258-rs9397585-rs685826) (Block II in Figure 2) and CC comprised of alleles of two *RGS17* SNPs (rs6931160-rs1281962) (Block III in Figure 2) were associated with one or more of these four dependence phenotypes (0.004≤*P*_obs_≤0.044); only the association of CCCC (potentially a risk haplotype) with OD withstood permutation-based multiple testing correction (*P*_empirical_ = 0.028). Detailed haplotype analysis results are presented in Additional file 1: Tables S3 and S4.

**Table 4:** **Association of*RGS17*haplotypes and four substance dependence (SD) traits**

**LD Blocks**	**Haplotypes**	**Phenotypes**	**Frequencies**	**χ**^**2**^	***P***_***obs***_	***P***_***emp***_
**(AAs)**			**(Case/control)**			
RGS17_I	GATTC	AD	0.053\ 0.032	5.09	0.024	0.319
	GATTC	CD	0.053\ 0.032	5.41	0.020	0.267
	GATTC	OD	0.067\ 0.032	9.25	0.002	0.026
	GATTC	MjD	0.057\ 0.032	5.95	0.015	0.178
	GTTCT	AD	0.015\ 0.029	5.83	0.016	0.222
	GTTCT	CD	0.016\ 0.029	5.44	0.020	0.265
	GTTCT	OD	0.010\ 0.029	6.65	0.010	0.106
	GTTCT	MjD	0.010\ 0.029	8.44	0.004	0.048
RGS17_II	CCT	OD	0.056\ 0.032	4.49	0.034	0.375
	TGT	OD	0.437\ 0.491	4.16	0.042	0.436
	TGT	MjD	0.440\ 0.491	4.35	0.037	0.405
RGS17_III	CC	MjD	0.528\ 0.472	5.35	0.021	0.240
**LD Blocks**	**Haplotypes**	**Phenotypes**	**Frequencies**	**χ**^**2**^	***P***_***obs***_	***P***_***emp***_
**(EAs)**			**(Case/Control)**			
RGS17_I	GTCTT	AD	0.211\ 0.258	5.75	0.017	0.159
	GTCTT	CD	0.206\ 0.258	7.14	0.008	0.070
	GTCTT	OD	0.210\ 0.258	5.56	0.018	0.169
RGS17_II	CCCC	CD	0.302\ 0.260	4.07	0.044	0.387
	CCCC	OD	0.324\ 0.260	8.26	0.004	0.028
	CCCC	MjD	0.309\ 0.260	3.98	0.046	0.345
RGS17_III	CC	OD	0.471\ 0.420	4.56	0.033	0.317

AD, alcohol dependence; CD, cocaine dependence; OD, opioid dependence; MjD, marijuana dependence.

*P*_obs_, observed *P* values using Pearson’s Chi-squared tests.

*P*_*emp*_, empirical *P* values using permutation-based tests for multiple testing corrections.

### *Transcription factor binding sites and correlation of genotypes with expression*

**Figure 3:**
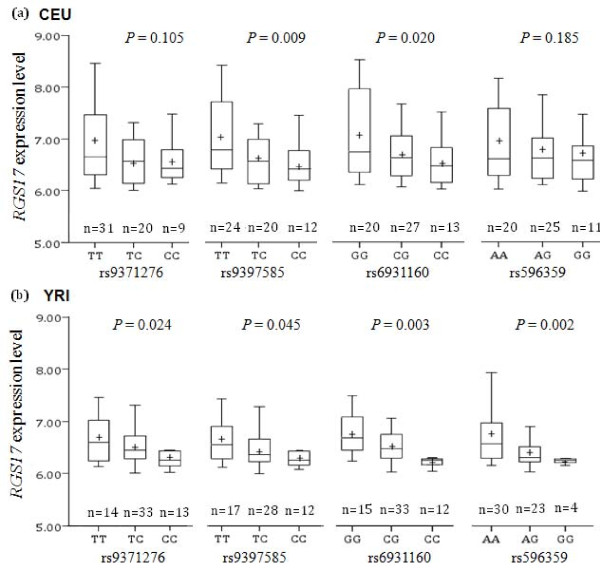
**Association of genotypes of four* RGS17 *SNPs and *RGS17 *mRNA expression levels.** The association between four *RGS17* SNPs (rs9371276, rs9397585, rs6931160 and rs596359, which were significantly associated with substance dependence) and *RGS17* mRNA expression levels was examined in 60 CEU (a) and 60 YRI (b) unrelated subjects. *P* values were calculated by linear regression analysis and adjusted by sex. The ends of the vertical lines indicate the minimum and maximum values. The lower hinge of the boxes indicates the 25^th^ percentile, the upper hinge of the boxes indicates the 75^th^ percentile, and the line in the boxes indicates the median value. “+” in the boxes refers to the mean expression level. X axis: *RGS17* SNP genotypes; Y axis: *RGS7* mRNA expression levels (mean ± SEM).

*RGS17* promoter SNP rs596359, which was strongly associated with multiple SD traits in both AAs and EAs, was predicted to be located in the binding site of transcription factor AML1a (core binding site: TGTGGT, corresponding to the G allele but not the A allele). Logistic regression analysis (assuming an additive model) indicated that genotypes of four *RGS17* SNPs (rs9371276, rs9397585, rs6931160, and rs596359, which were significantly associated with one or more SD traits), were significantly associated with *RGS17* mRNA expression levels in the YRI (rs9371276: T = −2.32, *P* = 0.024; rs9397585: T = −2.05, *P* = 0.045; rs6931160: T = 3.13, *P* = 0.003; rs596359: T = −3.25, *P* = 0.002) or the CEU subjects (rs9371276: T = −1.65, *P* = 0.105; rs9397585: T = −2.73, *P* = 0.009; rs6931160: T = 2.40, *P* = 0.020; rs596359: T = −1.34, *P* = 0.185) (Figure 3 and Additional file 1: Table S5. No significant association was observed between genotypes of the 13 *RGS17* SNPs and the expression level of the physically linked gene, *OPRM1* (Additional file 1: Table S6).

## Discussion

RGS17 (RGS-Z2) and RGS20 (RGS-Z1) are two members of the RGS-Rz subfamily of GTPase-activating proteins (GAP) that efficiently deactivate GalphazGTP subunits, and thereby turn off the signaling pathway of G protein-coupled receptors (GPCRs), including opioid receptors. Considering their intimate interactions with opioid receptors (mainly the mu-receptor) and their gene locations (*RGS17* is in the vicinity of *OPRM1* and *RGS20* is in the vicinity of *OPRK1*), the two genes (*RGS17* and *RGS20*) encoding them are both positional and functional susceptibility candidate loci for SD. We found that multiple *RGS17* SNPs were associated with multiple SD phenotypes in both AAs and EAs. However, none of the eight *RGS20* SNPs were associated with any of the four dependence traits.

Although variation in *RGS17* influences susceptibility to multiple dependence traits in both AAs and EAs, our results suggest that different mechanisms may be operative in some cases. SNPs rs596359 (in the promoter region) and rs6931160 (in intron 1) were associated with at least one of the four dependence traits in both populations. SNPs rs9397585, rs1933258 and rs9371276, which are all located in intron 1 and the same haplotype block (Block II) (Figure 2), were associated with one of the four dependence traits only in EAs. SNPs rs516557 (in intron 2) and rs545323 (in intron 4) were associated with one of the four dependence traits in only AAs (Table 3). Analyses of haplotypes harboring these SNPs supported the individual SNP findings. Haplotypes GATTC and GTTCT, containing alleles (underlined) of SNP rs545323 (Figure 2, haplotype Block I) were associated with OD and MjD, respectively, only in AAs, after permutation tests to correct for multiple comparisons. Haplotype CCCC, containing alleles (underlined) of the three SNPs (rs9397585, rs1933258 and rs9371276) located in intron 1 and haplotype Block II (Figure 2) was associated with OD only in EAs, after permutation tests to correct for multiple comparisons. These findings suggest that the population-specific associations were dependent on the location of variants in *RGS17*.

The most statistically significant result was obtained for SNP rs596359, which is located in the promoter region of *RGS17*. Chi-square tests, logistic regression analyses, and permutation tests all showed a positive association between SNP rs596359 and all four SD phenotypes in both populations (Table 3). Further, meta-analyses that combined data from both AAs and EAs showed that SNP rs596359 yielded an odds ratio from 1.28 to 1.33 for risk of all four SD traits (data not shown). Specifically, the G allele of SNP rs596359 was significantly more frequent in cases than in controls in both populations (Additional file 1: Tables S1 and S2). Thus, this promoter variant may increase the risk for SD by influencing *RGS17* transcription. To validate the functional effect of this promoter variant on *RGS17* transcription, we analyzed the correlation of rs596359 genotypes and *RGS17* mRNA expression levels in lymphoblastoid cell lines from both CEU and YRI subjects recruited for the HapMap project (http://hapmap.ncbi.nlm.nih.gov/). The G allele of SNP rs596359 showed a dose-related decrease in *RGS17* transcription by decreasing mRNA expression levels (Figure 3). Moreover, bioinformatic analyses indicated that substitution of the A allele for the G allele at rs596359 site generated a transcription binding site in the promoter region of *RGS17* for transcription factor AML1a. This transcription factor has a higher affinity for DNA-binding than AML1b, but lacks the putative transcriptional activation domain that is possessed by AML1b. Thus, AML1a dominantly suppresses the transcriptional activity exerted by AML1b [36]. Several other studies have demonstrated that AML1a inhibited erythroid or granulocytic differentiation [37,38]. Based on these findings, we would speculate that rs596359 G allele carriers have lower *RGS17* activity and thus greater synaptic neurotransmission and rewarding function mediated by GPCRs such as opioid receptors. To test this hypothesis, the influence of SNP rs596359 on *RGS17* promoter activity should be measured using other approaches (e.g., luciferase reporter gene assays).

None of the eight *RGS20* SNPs showed significant association with any of the four SD phenotypes in either AAs or EAs. There are three possible explanations for this lack of association. First, the *RGS20* SNPs selected for this study may have a minor or undetectable effect on SD. Fine-mapping of this gene could identify variants showing a stronger association with SD traits. Second, *RGS20* may have a weak effect on susceptibility to SD due to its being physically linked to *OPRK1*, which has a less important role than *OPRM1* (which is physically linked to *RGS17*) in mediating the rewarding effects of alcohol or drugs [17,19]. Third, similar to *OPRK1*, which mediates the psychotomimetic effects of some drugs [39], *RGS20* may mainly regulate other biological activities than SD. Further studies are warranted to determine whether *RGS20* is a susceptibility gene for SD.

The present study has several limitations. First, our finding is limited by the relatively small size of the control sample. Moreover, we did not control for prior genotyping performed on this sample in multiple testing corrections because we were concerned that overly conservative results might be obtained. Second, SD frequently co-occurs with Axis I disorders (e.g., depression and anxiety disorders) and Axis II disorders (i.e., personality disorders). Thus, our findings of an association between *RGS17* variants and SD may be cofounded by comorbid disorders. Third, given the close relationship between the RGS-Rz (*RGS17* and *RGS20*) and opioid receptor (*OPRM1* and *OPRK1,* respectively) genes, gene-gene interaction analyses should be conducted. We would speculate that strong gene-gene interaction effects (e.g., of *OPRM1* and *RGS17*) on SD would be detectable. Even though variation at *RGS20* did not show significant association with SD in individual gene analysis, interaction effects of that gene with *OPRM1* or *OPRK1* on SD risk may exist. Fourth, in this study, we ignored polymorphisms in exonic regions because they are rare in the genes examined. There is only one known SNP rs2295230 (synonymous) in *RGS17* exon 2 that had a minor allele frequency greater than 5% in AA and EA populations. Exonic SNP rs2295230 is in tight LD with intronic SNP rs9371276 (which was included in this study) (CEU: D’ = 0.96, r^2^ =0.92; YRI: D’ = 0.86, r^2^ = 0.54, using genotyping data from the 1000 Genomes Project). It is also situated close to SNP rs2295230. Thus, exonic SNP rs2295230 was not considered in the present study. As we know, rare variants in coding regions may have a larger impact on disease risk (in the few individuals who carry them) than common non-coding variants (which may have a greater impact at the population level). Recent genome-wide association studies using common genetic variants have identified specific loci and/or genomic regions that contribute to the etiology of certain disorders. However, only a small proportion of the heritability of complex disorders, such as SD, can be accounted for by common variants [40,41]. Therefore, it is necessary to sequence exons of target genes (such as *RGS17* and *RGS20*) or perform exomic sequencing using next-generation sequencing technology to identify new rare variants and analyze their association with SD. Fifth, given the incomplete penetrance of susceptibility genes for alcohol or drug dependence in monozygotic twins [42], epigenetic mechanisms should be studied to determine their contribution to SD risk. Altered DNA methylation levels in a number of genes (e.g., *OPRM1*) have been found in patients with alcohol or opioid dependence [43,44]. Altered methylation of *RGS17* and *RGS20* (especially in their promoter regions) could increase the risk for SD. Therefore, epigenetic studies may provide further evidence about the role of *RGS17* and *RGS20* in the etiology of SD.

## Conclusions

In summary, we found that *RGS17* polymorphisms were associated with multiple SD phenotypes in both AA and EA populations. Our findings suggest that lower transcription levels of *RSG17* due to certain genetic variants (e.g., the promoter SNP rs596356) may modulate the reinforcing effects of alcohol or drugs that are mediated by GPCRs such as opioid receptors and thus influence the vulnerability to SD. Given the fact that *RGS17* is significantly expressed in striatal regions including the nucleus accumbens and putamen [45], if our findings are validated, *RGS17* and its protein product could be good targets for medications to treat SD.

## Abbreviations

AD, CD, OD or MjD: Alcohol, cocaine, opioid or marijuana dependence; AIM: Ancestry informative marker; CMH: The Cochran-Mantel-Haenszel (CMH) test; GAP: GTPase-activating protein; GEO: The Gene Expression Omnibus; GPCR: G protein-coupled receptor; HWE: Hardy-Weinberg equilibrium; OPRM1: The μ-opioid receptor (MOR) gene; OPRK1: The κ-opioid receptor (KOR) gene; RGS: Regulator of G-protein signaling protein; SD: Substance (alcohol or drug) dependence; SNP: Single nucleotide polymorphism; TESS: The Transcription Element Search System; TF: Transcription factor.

## Competing interests

Dr. Kranzler has received compensation for professional services from the National Institutes of Health (NIAAA and NIDA) and for academic lectures and editorial functions in various scientific venues (including the ACNP). Dr. Kranzler has had consulting arrangements with the following pharmaceutical companies: Alkermes, Gilead, GlaxoSmithKline, Lilly, Lundbeck, Pfizer, and Roche. Dr. Anton has had consulting agreements with the following companies: Eli Lilly, GlaxoSmithKline, Alkermes, Lundbeck, and Roche. Drs. Kranzler and Anton also receive support from the Alcohol Clinical Trials Initiative (ACTIVE), which Eli Lilly, Schering Plough, Lundbeck, Alkermes, GlaxoSmithKline, Abbott, and Johnson & Johnson support. Drs. Kranzler and Anton report research support from Merck and Dr. Anton from Eli Lilly. Dr. Gelernter reports that he has received compensation for professional services in the previous three years from the following entities: Yale University School of Medicine, Veterans Affairs Healthcare System (VA), and the National Institutes of Health (NIAAA, NIDA, and NIMH), and related to academic lectures and editorial functions in various scientific venues (including the ACNP). Other authors have no conflict of interest to report.

## Authors' contributions

HZ took part in planning, designing and conducting the experiments, collected the data, performed the data analysis and drafted the manuscript. FW helped performing the data analysis and drafting the manuscript. HRK, RFA and JG contributed to sample collection and helped draft the manuscript. All authors read and approved the final manuscript.

## Supplementary Material

Additional file 1 Supplementary materials Click here for file
