# Supplementary material for: Variation in regulator of G-protein signaling 17 gene (RGS17) is associated with multiple substance dependence diagnoses
Source: Behav Brain Funct. 2012 May 16;8:23. doi: 10.1186/1744-9081-8-23 (PMC3406967; doi:10.1186/1744-9081-8-23)
Supplement: Additional file 1 — Supplementary materials [file 1744-9081-8-23-S1.doc]

**Supplementary Materials**

**Table S1** Association analysis of 13 *RGS17* SNPs with four substance dependence phenotypes

**Table S2** Association analysis of eight *RGS20* SNPs with four substance dependence phenotypes

**Table S3** Haplotype-based association analysis *RGS17* SNPs in African Americans

**Table S4** Haplotype-based as**s**ociation analysis *RGS17* SNPs in European Americans

**Table S5** Association of genotypes of *RGS17* SNPs with *RGS17* expression levels

**Table S6** Association of genotypes of *RGS17* SNPs with *OPRM1* expression levels

**Figure S1** Regional plots of the association between 13 *RGS17* SNPs and substance dependence in African Americans (AAs)

**Figure S2** Regional plots of the association between 13 *RGS17* SNPs and substance dependence in European Americans (EAs)

**Table S1 Association analysis of 13 *RGS17* SNPs with four substance dependence phenotypes**

| **Traits** | **SNPs** | **Race** | **RA** | **Frequency** | ***Х^2^*** | ***P_obs_*** | ***OR (95%CI)*** | ***P_adj_*** | ***OR (95%CI)*** | ***P_emp_*** |
| --- | --- | --- | --- | --- | --- | --- | --- | --- | --- | --- |
| AD | rs9397578 | AA | A | 0.28\0.27 | 0.71 | 0.400 | 1.09(0.89-1.32) | 0.351 | 1.10(0.91-1.33) | 0.398 |
|  |  | EA | A | 0.26\0.25 | 0.2 | 0.653 | 1.05(0.85-1.30) | 0.705 | 1.04(0.84-1.29) | 0.665 |
|  | rs7750874 | AA | A | 0.3\0.29 | 0.06 | 0.807 | 1.02(0.85-1.24) | 0.846 | 1.02(0.84-1.24) | 0.784 |
|  |  | EA | A | 0.34\0.34 | 0.01 | 0.915 | 1.01(0.83-1.23) | 0.893 | 1.01(0.83-1.24) | 0.921 |
|  | rs503366 | AA | C | 0.48\0.45 | 1.05 | 0.306 | 1.10(0.92-1.30) | 0.281 | 1.10(0.92-1.31) | 0.287 |
|  |  | EA | C | 0.48\0.51 | 2.24 | 0.135 | 0.87(0.72-1.04) | 0.108 | 0.86(0.71-1.03) | 0.120 |
|  | rs610614 | AA | C | 0.43\0.42 | 0.03 | 0.856 | 1.02(0.85-1.22) | 0.826 | 1.02(0.85-1.22) | 0.854 |
|  |  | EA | C | 0.3\0.29 | 0.23 | 0.632 | 1.05(0.86-1.29) | 0.367 | 1.04(0.85-1.28) | 0.636 |
|  | rs545323 | AA | C | 0.06\0.03 | 6.7 | **0.010** | 1.96(1.17-3.30) | **0.013** | 1.96(1.16-3.32) | **0.010** |
|  |  | EA | C | 0.33\0.32 | 0.23 | 0.629 | 1.05(0.86-1.28) | 0.598 | 1.06(0.86-1.29) | 0.630 |
|  | rs516557 | AA | T | 0.49\0.43 | 4.78 | **0.029** | 1.25(1.02-1.52) | **0.035** | 1.23(1.02-1.50) | **0.018** |
|  |  | EA | C | 0.48\0.47 | 0.23 | 0.628 | 1.05(0.87-1.26) | 0.714 | 1.04(0.86-1.24) | 0.66 |
|  | rs9371276 | AA | T | 0.46\0.51 | 4.08 | **0.043** | 0.84(0.70-0.99) | **0.045** | 0.84(0.71-1.00) | 0.053 |
|  |  | EA | C | 0.30\0.27 | 2.83 | 0.093 | 1.19(0.97-1.47) | 0.113 | 1.18(0.96-1.45) | 0.092 |
|  | rs1933258 | AA | G | 0.48\0.51 | 2.33 | 0.127 | 0.87(0.73-1.04) | 0.135 | 0.88(0.74-1.04) | 0.149 |
|  |  | EA | C | 0.31\0.27 | 3.32 | 0.068 | 1.21(0.99-1.50) | 0.072 | 1.21(0.98-1.50) | 0.061 |
|  | rs9397585 | AA | C | 0.49\0.47 | 1.29 | 0.257 | 1.10(0.93-1.32) | 0.258 | 1.11(0.93-1.32) | 0.267 |
|  |  | EA | C | 0.37\0.35 | 0.95 | 0.331 | 1.10(0.91-1.33) | 0.245 | 1.13(0.92-1.37) | 0.345 |
|  | rs685826 | AA | T | 0.46\0.47 | 0.64 | 0.423 | 0.93(0.78-1.11) | 0.468 | 0.94(0.79-1.11) | 0.450 |
|  |  | EA | C | 0.44\0.44 | 0.00 | 0.963 | 1.00(0.82-1.20) | 0.753 | 1.03(0.85-1.25) | 0.968 |
|  | rs6931160 | AA | G | 0.49\0.53 | 3.55 | 0.060 | 0.85(0.71-1.01) | 0.080 | 0.86(0.72-1.02) | 0.054 |
|  |  | EA | C | 0.43\0.42 | 0.29 | 0.591 | 1.05(0.87-1.27) | 0.532 | 1.06(0.88-1.28) | 0.596 |
|  | rs1281962 | AA | G | 0.24\0.26 | 1.29 | 0.257 | 0.89(0.73-1.09) | 0.259 | 0.89(0.73-1.09) | 0.276 |
|  |  | EA | G | 0.46\0.47 | 0.2 | 0.659 | 0.96(0.80-1.15) | 0.523 | 0.94(0.78-1.13) | 0.660 |
|  | rs596359 | AA | G | 0.35\0.29 | 7.82 | **0.005** | 1.31(1.08-1.58) | **0.008** | 1.29(1.07-1.56) | **0.005** |
|  |  | EA | G | 0.50\0.44 | 6.37 | **0.012** | 1.27(1.05-1.52) | **0.011** | 1.27(1.06-1.53) | **0.014** |
| CD | rs9397578 | AA | A | 0.27\0.27 | 0.09 | 0.764 | 1.03(0.85-1.25) | 0.763 | 1.03(0.85-1.25) | 0.790 |
|  |  | EA | A | 0.25\0.25 | 0.01 | 0.942 | 1.01(0.81-1.25) | 0.927 | 1.01(0.82-1.25) | 0.965 |
|  | rs7750874 | AA | A | 0.30\0.29 | 0.04 | 0.835 | 1.02(0.85-1.23) | 0.793 | 1.03(0.85-1.24) | 0.840 |
|  |  | EA | A | 0.35\0.34 | 0.26 | 0.607 | 1.05(0.87-1.28) | 0.684 | 1.04(0.85-1.27) | 0.599 |
|  | rs503366 | AA | C | 0.47\0.45 | 0.45 | 0.505 | 1.06(0.89-1.26) | 0.505 | 1.06(0.89-1.25) | 0.523 |
|  |  | EA | C | 0.47\0.51 | 4.02 | **0.045** | 0.83(0.69-1.00) | **0.042** | 0.82(0.69-0.99) | 0.056 |
|  | rs610614 | AA | C | 0.42\0.42 | 0.05 | 0.825 | 0.98(0.82-1.17) | 0.818 | 0.98(0.82-1.17) | 0.834 |
|  |  | EA | C | 0.31\0.29 | 0.3 | 0.582 | 1.06(0.86-1.30) | 0.655 | 1.05(0.85-1.28) | 0.572 |
|  | rs545323 | AA | C | 0.05\0.03 | 6.74 | **0.009** | 1.95(1.17-3.25) | **0.015** | 1.95(1.16-3.28) | **0.009** |
|  |  | EA | C | 0.34\0.32 | 0.98 | 0.321 | 1.10(0.91-1.34) | 0.347 | 1.10(0.90-1.35) | 0.321 |
|  | rs516557 | AA | T | 0.48\0.43 | 3.53 | 0.06 | 1.21(0.99-1.47) | 0.084 | 1.18(0.98-1.40) | 0.080 |
|  |  | EA | C | 0.48\0.47 | 0.53 | 0.465 | 1.07(0.88-1.29) | 0.524 | 1.06(0.88-1.28) | 0.490 |
|  | rs9371276 | AA | T | 0.47\0.51 | 2.44 | 0.118 | 0.87(0.74-1.04) | 0.117 | 0.87(0.74-1.04) | 0.109 |
|  |  | EA | C | 0.31\0.27 | 3.98 | **0.046** | 1.23(1.00-1.51) | 0.063 | 1.22(0.99-1.49) | 0.053 |
|  | rs1933258 | AA | G | 0.49\0.51 | 1.26 | 0.262 | 0.91(0.76-1.08) | 0.275 | 0.91(0.77-1.08) | 0.275 |
|  |  | EA | C | 0.31\0.27 | 4.54 | **0.033** | 1.25(1.02-1.54) | **0.040** | 1.25(1.01-1.54) | **0.037** |
|  | rs9397585 | AA | C | 0.49\0.47 | 1.10 | 0.294 | 1.10(0.92-1.30) | 0.307 | 1.09(0.92-1.30) | 0.295 |
|  |  | EA | C | 0.38\0.35 | 1.40 | 0.236 | 1.12(0.93-1.36) | 0.202 | 1.14(0.93-1.39) | 0.233 |
|  | rs685826 | AA | T | 0.46\0.47 | 0.77 | 0.381 | 0.93(0.78-1.10) | 0.406 | 0.93(0.79-1.10) | 0.399 |
|  |  | EA | C | 0.44\0.45 | 0.03 | 0.874 | 0.99(0.82-1.19) | 0.843 | 1.02(0.84-1.24) | 0.876 |
|  | rs6931160 | AA | G | 0.49\0.53 | 4.56 | **0.033** | 0.83(0.70-0.99) | **0.036** | 0.83(0.70-0.99) | **0.028** |
|  |  | EA | C | 0.44\0.42 | 1.01 | 0.315 | 1.10(0.91-1.32) | 0.371 | 1.09(0.90-1.32) | 0.605 |
|  | rs1281962 | AA | G | 0.24\0.26 | 1.02 | 0.313 | 0.90(0.75-1.10) | 0.313 | 0.90(0.74-1.10) | 0.313 |
|  |  | EA | G | 0.45\0.47 | 0.36 | 0.550 | 0.95(0.79-1.14) | 0.513 | 0.94(0.78-1.13) | 0.557 |
|  | rs596359 | AA | G | 0.34\0.29 | 6.77 | **0.009** | 1.28(1.06-1.54) | **0.011** | 1.27(1.06-1.53) | **0.009** |
|  |  | EA | G | 0.51\0.44 | 6.91 | **0.009** | 1.28(1.06-1.54) | **0.008** | 1.29(1.07-1.56) | **0.006** |
| OD | rs9397578 | AA | A | 0.28\0.27 | 0.24 | 0.623 | 1.06(0.84-1.35) | 0.641 | 1.06(0.84-1.33) | 0.644 |
|  |  | EA | A | 0.25\0.25 | 0.06 | 0.802 | 1.03(0.82-1.28) | 0.732 | 1.04(0.84-1.29) | 0.838 |
|  | rs7750874 | AA | A | 0.32\0.29 | 1.47 | 0.226 | 1.15(0.92-1.45) | 0.183 | 1.18(0.93-1.49) | 0.219 |
|  |  | EA | A | 0.34\0.34 | 0.13 | 0.721 | 1.04(0.85-1.27) | 0.792 | 1.03(0.84-1.27) | 0.716 |
|  | rs503366 | AA | C | 0.46\0.45 | 0.00 | 0.954 | 1.01(0.81-1.24) | 0.965 | 1.01(0.81-1.24) | 0.964 |
|  |  | EA | C | 0.48\0.51 | 2.28 | 0.131 | 0.85(0.71-1.05) | 0.137 | 0.86(0.71-0.05) | 0.140 |
|  | rs610614 | AA | C | 0.42\0.42 | 0.04 | 0.840 | 0.98(0.78-1.22) | 0.800 | 0.97(0.78-1.21) | 0.851 |
|  |  | EA | C | 0.31\0.29 | 0.82 | 0.364 | 1.10(0.89-1.36) | 0.405 | 1.09(0.89-1.35) | 0.377 |
|  | rs545323 | AA | C | 0.07\0.03 | 11.27 | **0.001** | 2.55(1.45-4.49) | **0.002** | 2.51(1.41-4.46) | **0.003** |
|  |  | EA | C | 0.34\0.32 | 0.8 | 0.371 | 1.10(0.90-1.35) | 0.364 | 1.10(0.89-1.36) | 0.381 |
|  | rs516557 | AA | T | 0.46\0.43 | 1.25 | 0.265 | 1.14(0.9-1.44) | 0.325 | 1.12(0.89-1.41) | 0.271 |
|  |  | EA | C | 0.48\0.47 | 0.15 | 0.694 | 1.04(0.86-1.26) | 0.694 | 1.04(0.86-1.26) | 0.703 |
|  | rs9371276 | AA | T | 0.45\0.51 | 3.96 | 0.047 | 0.81(0.65-1.00) | 0.043 | 0.81(0.65-0.99) | 0.051 |
|  |  | EA | C | 0.33\0.27 | 7.47 | **0.006** | 1.34(1.09-1.66) | **0.008** | 1.33(1.08-1.63) | **0.008** |
|  | rs1933258 | AA | G | 0.47\0.51 | 2.09 | 0.148 | 0.85(0.69-1.06) | 0.125 | 0.85(0.69-1.05) | 0.166 |
|  |  | EA | C | 0.33\0.27 | 8.51 | **0.004** | 1.37(1.11-1.70) | **0.004** | 1.36(1.10-1.68) | **0.005** |
|  | rs9397585 | AA | C | 0.49\0.47 | 0.81 | 0.367 | 1.10(0.89-1.36) | 0.335 | 1.11(0.90-1.38) | 0.362 |
|  |  | EA | C | 0.40\0.35 | 4.39 | **0.036** | 1.24(1.01-1.51) | **0.020** | 1.27(1.04-1.56) | **0.028** |
|  | rs685826 | AA | T | 0.45\0.47 | 0.95 | 0.331 | 0.90(0.73-1.11) | 0.326 | 0.90(0.73-1.11) | 0.322 |
|  |  | EA | C | 0.47\0.44 | 1.27 | 0.260 | 1.12(0.92-1.36) | 0.149 | 1.16(0.95-1.41) | 0.272 |
|  | rs6931160 | AA | G | 0.51\0.53 | 1.02 | 0.314 | 0.90(0.73-1.11) | 0.356 | 0.91(0.73-1.12) | 0.321 |
|  |  | EA | C | 0.48\0.42 | 5.14 | **0.023** | 1.25(1.03-1.51) | **0.019** | 1.26(1.04-1.54) | **0.018** |
|  | rs1281962 | AA | G | 0.24\0.26 | 0.6 | 0.438 | 0.91(0.71-1.16) | 0.357 | 0.89(0.70-1.14) | 0.470 |
|  |  | EA | G | 0.43\0.47 | 2.79 | 0.095 | 0.85(0.70-1.03) | 0.073 | 0.84(0.69-1.02) | 0.087 |
|  | rs596359 | AA | G | 0.35\0.29 | 6.5 | **0.011** | 1.34(1.07-1.69) | **0.023** | 1.31(1.04-1.63) | **0.019** |
|  |  | EA | G | 0.5\0.44 | 5.54 | **0.019** | 1.26(1.04-1.52) | **0.010** | 1.29(1.06-1.57) | **0.013** |
| MjD | rs9397578 | AA | A | 0.26\0.27 | 0.09 | 0.765 | 0.97(0.78-1.21) | 0.770 | 0.97(0.78-1.20) | 0.775 |
|  |  | EA | A | 0.25\0.25 | 0.00 | 0.944 | 0.99(0.77-1.28) | 0.884 | 0.97(0.76-1.27) | 0.945 |
|  | rs7750874 | AA | A | 0.32\0.29 | 1.13 | 0.288 | 1.12(0.91-1.39) | 0.284 | 1.13(0.91-1.41) | 0.269 |
|  |  | EA | A | 0.33\0.34 | 0.14 | 0.704 | 0.96(0.74-1.20) | 0.664 | 0.95(0.75-1.20) | 0.684 |
|  | rs503366 | AA | C | 0.45\0.45 | 0.11 | 0.740 | 0.97(0.80-1.18) | 0.774 | 0.97(0.80-1.18) | 0.717 |
|  |  | EA | C | 0.47\0.51 | 2.68 | 0.102 | 0.83(0.67-1.04) | 0.104 | 0.83(0.67-1.04) | 0.093 |
|  | rs610614 | AA | C | 0.4\0.42 | 1.03 | 0.374 | 0.91(0.74-1.12) | 0.400 | 0.92(0.75-1.12) | 0.379 |
|  |  | EA | C | 0.31\0.29 | 0.29 | 0.590 | 1.07(0.84-1.36) | 0.674 | 1.05(0.83-1.34) | 0.605 |
|  | rs545323 | AA | C | 0.06\0.03 | 7.3 | **0.007** | 2.10(1.21-3.64) | **0.009** | 2.10(1.20-3.67) | **0.009** |
|  |  | EA | C | 0.31\0.32 | 0.00 | 0.956 | 0.99(0.79-1.26) | 0.968 | 1.00(0.78-1.26) | 0.962 |
|  | rs516557 | AA | T | 0.48\0.43 | 3.28 | 0.070 | 1.22(0.98-1.52) | 0.177 | 1.22(0.98-1.51) | 0.069 |
|  |  | EA | C | 0.48\0.47 | 0.20 | 0.658 | 1.05(0.84-1.31) | 0..724 | 1.04(0.84-1.29) | 0.682 |
|  | rs9371276 | AA | T | 0.46\0.51 | 4.21 | **0.040** | 0.82(0.67-0.99) | **0.038** | 0.82(0.67-0.99) | 0.055 |
|  |  | EA | C | 0.33\0.27 | 5.11 | **0.024** | 1.32(1.04-1.62) | **0.053** | 1.26(1.00-1.58) | **0.032** |
|  | rs1933258 | AA | G | 0.47\0.51 | 2.80 | 0.094 | 0.85(0.70-1.03) | 0.089 | 0.85(0.70-1.03) | 0.121 |
|  |  | EA | C | 0.33\0.27 | 5.63 | **0.018** | 1.34(1.05-1.70) | **0.036** | 1.29(1.02-1.63) | **0.024** |
|  | rs9397585 | AA | C | 0.50\0.47 | 1.38 | 0.241 | 1.12(0.93-1.36) | 0.224 | 1.13(0.93-1.37) | 0.266 |
|  |  | EA | C | 0.41\0.35 | 4.36 | **0.037** | 1.27(1.02-1.59) | **0.035** | 1.28(1.02-1.61) | **0.030** |
|  | rs685826 | AA | T | 0.45\0.47 | 0.66 | 0.418 | 0.92(0.76-1.12) | 0.436 | 0.93(0.76-1.12) | 0.442 |
|  |  | EA | C | 0.47\0.44 | 1.14 | 0.285 | 1.13(0.90-1.40) | 0.209 | 1.15(0.92-1.44) | 0.275 |
|  | rs6931160 | AA | G | 0.47\0.53 | 6.35 | **0.012** | 0.78(0.64-0.95) | **0.015** | 0.79(0.65-0.95) | **0.016** |
|  |  | EA | C | 0.45\0.42 | 0.79 | 0.377 | 1.10(0.89-1.37) | 0.413 | 1.10(0.88-1.36) | 0.392 |
|  | rs1281962 | AA | G | 0.23\0.26 | 2.00 | 0.158 | 0.85(0.68-1.07) | 0.138 | 0.84(0.67-1.06) | 0.159 |
|  |  | EA | G | 0.44\0.47 | 1.03 | 0.309 | 0.89(0.72-1.11) | 0.334 | 0.90(0.73-1.12) | 0.322 |
|  | rs596359 | AA | G | 0.35\0.29 | 8.05 | **0.005** | 1.35(1.10-1.67) | **0.008** | 1.33(1.08-1.63) | **0.006** |
|  |  | EA | G | 0.51\0.44 | 5.70 | **0.017** | 1.30(1.05-1.62) | **0.015** | 1.31(1.05-1.63) | **0.013** |
|  | RA, reference allele; Frequency, RA frequency in case/control; *P_obs_*, observed *P* values using the Pearson’s Chi-square test; *P_adj_*, adjusted *P* values calculated by multivariate logistic regression after adjustment by sex, age and ancestry proportion, under the additive model; *P_emp_*, empirical *P* values calculated from 10,000 times of permutations test; OR, odds ratio; 95%CI, 95% confidence interval. | | | | | | | | | |

**Table S2 Association analysis of eight *RGS20* SNPs with four substance dependence phenotypes**

| **Traits** | **SNPs** | **Race** | **RA** | **Frequency** | ***Х^2^*** | ***P_obs_*** | ***OR (95%CI)*** | ***P_adj_*** | ***OR (95%CI)*** | ***P_emp_*** |
| --- | --- | --- | --- | --- | --- | --- | --- | --- | --- | --- |
| AD | rs1384797 | AA | G | 0.41\0.41 | 0.00 | 0.958 | 1.00(0.83-1.19) | 0.973 | 1.00(0.84-1.16) | 0.965 |
|  |  | EA | G | 0.02\0.02 | 0.04 | 0.845 | 1.06(0.57-1.99) | 0.797 | 0.92(0.48-1.77) | 0.857 |
|  | rs2220093 | AA | A | 0.35\0.36 | 0.01 | 0.938 | 0.99(0.83-1.19) | 0.856 | 0.98(0.82-1.18) | 0.934 |
|  |  | EA | G | 0.10\0.11 | 0.31 | 0.58 | 0.92(0.68-1.24) | 0.537 | 0.91(0.66-1.24) | 0.600 |
|  | rs1483537 | AA | G | 0.36\0.36 | 0.01 | 0.941 | 0.99(0.83-1.19) | 0.989 | 1.00(0.83-1.20) | 0.935 |
|  |  | EA | G | 0.01\0.01 | 0.01 | 0.937 | 1.05(0.32-3.50) | 0.816 | 0.86(0.23-3.19) | 0.931 |
|  | rs7824575 | AA | G | 0.24\0.24 | 0.00 | 0.986 | 1.00(0.81-1.23) | 0.878 | 0.98(0.80-1.21) | 0.999 |
|  |  | EA | A | 0.27\0.24 | 1.6 | 0.206 | 1.15(0.93-1.42) | 0.271 | 1.13(0.91-1.40) | 0.221 |
|  | rs2128821 | AA | C | 0.42\0.44 | 0.65 | 0.421 | 0.93(0.78-1.11) | 0.405 | 0.93(0.78-1.11) | 0.435 |
|  |  | EA | G | 0.26\0.24 | 0.94 | 0.334 | 1.11(0.90-1.37) | 0.371 | 1.10(0.89-1.37) | 0.334 |
|  | rs9298496 | AA | C | 0.38\0.37 | 0.14 | 0.707 | 1.04(0.86-1.24) | 0.715 | 1.03(0.86-1.24) | 0.690 |
|  |  | EA | C | 0.34\0.33 | 0.04 | 0.837 | 1.02(0.84-1.25) | 0.896 | 1.01(0.83-1.23) | 0.847 |
|  | rs6981243 | AA | A | 0.41\0.44 | 1.87 | 0.171 | 0.88(0.74-1.06) | 0.146 | 0.88(0.73-1.05) | 0.150 |
|  |  | EA | C | 0.42\0.39 | 1.6 | 0.205 | 1.13(0.93-1.37) | 0.276 | 1.11(0.92-1.34) | 0.231 |
|  | rs7009781 | AA | T | 0.27\0.26 | 0.44 | 0.508 | 1.07(0.88-1.31) | 0.633 | 1.05(0.86-1.28) | 0.500 |
|  |  | EA | C | 0.17\0.17 | 0.08 | 0.775 | 0.97(0.76-1.23) | 0.756 | 0.96(0.76-1.23) | 0.796 |
| CD | rs1384797 | AA | G | 0.39\0.41 | 0.71 | 0.399 | 0.93(0.78-1.10) | 0.460 | 0.94(0.79-1.11) | 0.397 |
|  |  | EA | G | 0.02\0.02 | 0.03 | 0.857 | 1.06(0.57-1.97) | 0.604 | 0.84(0.44-1.62) | 0.878 |
|  | rs2220093 | AA | A | 0.37\0.36 | 0.22 | 0.640 | 1.04(0.87-1.25) | 0.687 | 1.04(0.87-1.24) | 0.643 |
|  |  | EA | G | 0.09\0.11 | 0.84 | 0.360 | 0.87(0.64-1.17) | 0.269 | 0.84(0.61-1.15) | 0.363 |
|  | rs1483537 | AA | G | 0.35\0.36 | 0.01 | 0.930 | 0.99(0.83-1.19) | 0.980 | 1.00(0.84-1.20) | 0.927 |
|  |  | EA | G | 0.01\0.01 | 0.00 | 0.990 | 0.99(0.30-3.31) | 0.769 | 0.82(0.22-3.11) | 0.977 |
|  | rs7824575 | AA | G | 0.24\0.24 | 0.05 | 0.817 | 1.02(0.84-1.25) | 0.910 | 1.01(0.83-1.24) | 0.823 |
|  |  | EA | A | 0.27\0.24 | 1.34 | 0.246 | 1.13(0.92-1.40) | 0.378 | 1.10(0.89-1.38) | 0.255 |
|  | rs2128821 | AA | C | 0.42\0.44 | 0.85 | 0.356 | 0.92(0.77-1.10) | 0.335 | 0.92(0.77-1.09) | 0.367 |
|  |  | EA | G | 0.26\0.24 | 0.57 | 0.452 | 1.09(0.88-1.34) | 0.544 | 1.07(0.86-1.33) | 0.430 |
|  | rs9298496 | AA | C | 0.38\0.37 | 0.29 | 0.593 | 1.05(0.88-1.26) | 0.622 | 1.05(0.88-1.25) | 0.604 |
|  |  | EA | C | 0.33\0.33 | 0.00 | 0.992 | 1.00(0.82-1.22) | 0.835 | 0.98(0.80-1.20) | 1.000 |
|  | rs6981243 | AA | A | 0.42\0.44 | 0.80 | 0.371 | 0.92(0.78-1.10) | 0.337 | 0.92(0.77-1.09) | 0.353 |
|  |  | EA | C | 0.41\0.39 | 0.74 | 0.388 | 1.09(0.90-1.31) | 0.430 | 1.08(0.89-1.30) | 0.442 |
|  | rs7009781 | AA | T | 0.27\0.26 | 0.93 | 0.335 | 1.10(0.91-1.34) | 0.469 | 1.07(0.88-1.31) | 0.352 |
|  |  | EA | C | 0.18\0.17 | 0.03 | 0.859 | 1.02(0.80-1.30) | 0.955 | 1.01(0.79-1.28) | 0.870 |
| OD | rs1384797 | AA | G | 0.39\0.41 | 0.64 | 0.425 | 0.91(0.74-1.14) | 0.551 | 0.94(0.75-1.17) | 0.426 |
|  |  | EA | G | 0.02\0.02 | 0.18 | 0.671 | 0.86(0.44-1.69) | 0.449 | 0.76(0.38-1.54) | 0.628 |
|  | rs2220093 | AA | A | 0.37\0.36 | 0.12 | 0.726 | 1.04(0.83-1.30) | 0.920 | 1.01(0.81-1.26) | 0.744 |
|  |  | EA | G | 0.09\0.11 | 1.25 | 0.365 | 0.84(0.61-1.15) | 0.243 | 0.82(0.60-1.14) | 0.280 |
|  | rs1483537 | AA | G | 0.37\0.36 | 0.12 | 0.730 | 1.04(0.83-1.30) | 0.666 | 1.05(0.84-1.31) | 0.735 |
|  |  | EA | G | 0.01\0.01 | 0.13 | 0.718 | 1.25(0.37-4.16) | 0.994 | 0.99(0.26-3.82) | 0.772 |
|  | rs7824575 | AA | G | 0.23\0.24 | 0.09 | 0.767 | 0.96(0.75-1.24) | 0.634 | 0.94(0.73-1.21) | 0.763 |
|  |  | EA | A | 0.27\0.24 | 1.51 | 0.219 | 1.15(0.92-1.43) | 0.224 | 1.18(0.92-1.46) | 0.149 |
|  | rs2128821 | AA | C | 0.42\0.44 | 0.51 | 0.474 | 0.92(0.74-1.15) | 0.515 | 0.93(0.76-1.15) | 0.488 |
|  |  | EA | G | 0.27\0.24 | 1.29 | 0.257 | 1.14(0.91-1.41) | 0.269 | 1.14(0.91-1.43) | 0.248 |
|  | rs9298496 | AA | C | 0.38\0.37 | 0.17 | 0.678 | 1.05(0.84-1.31) | 0.742 | 1.04(0.83-1.30) | 0.673 |
|  |  | EA | C | 0.33\0.33 | 0.00 | 0.992 | 1.00(0.81-1.23) | 0.885 | 0.98(0.80-1.22) | 1.000 |
|  | rs6981243 | AA | A | 0.43\0.44 | 0.13 | 0.720 | 0.96(0.77-1.19) | 0.600 | 0.94(0.76-1.17) | 0.741 |
|  |  | EA | C | 0.43\0.39 | 1.70 | 0.193 | 1.14(0.94-1.39) | 0.241 | 1.13(0.92-1.37) | 0.188 |
|  | rs7009781 | AA | T | 0.26\0.26 | 0.04 | 0.842 | 1.03(0.80-1.31) | 0.869 | 0.98(0.77-1.25) | 0.854 |
|  |  | EA | C | 0.18\0.17 | 0.23 | 0.632 | 1.06(0.83-1.37) | 0.62 | 1.07(0.83-1.37) | 0.640 |
| MjD | rs1384797 | AA | G | 0.39\0.41 | 0.46 | 0.500 | 0.93(0.77-1.14) | 0.608 | 0.95(0.78-1.16) | 0.506 |
|  |  | EA | G | 0.02\0.02 | 0.00 | 0.957 | 1.02(0.49-2.13) | 0.724 | 0.87(0.40-1.90) | 0.996 |
|  | rs2220093 | AA | A | 0.36\0.36 | 0.01 | 0.923 | 1.01(0.82-1.24) | 0.938 | 0.99(0.81-1.22) | 0.932 |
|  |  | EA | G | 0.08\0.11 | 1.82 | 0.177 | 0.78(0.54-1.12) | 0.135 | 0.75(0.51-1.10) | 0.184 |
|  | rs1483537 | AA | G | 0.37\0.36 | 0.28 | 0.598 | 1.06(0.86-1.30) | 0.510 | 1.07(0.87-1.32) | 0.606 |
|  |  | EA | G | 0.01\0.01 | 0.24 | 0.621 | 1.39(0.37-5.21) | 0.735 | 1.29(0.30-5.47) | 0.651 |
|  | rs7824575 | AA | G | 0.24\0.24 | 0.02 | 0.902 | 0.99(0.78-1.24) | 0.740 | 0.96(0.76-1.21) | 0.898 |
|  |  | EA | A | 0.26\0.24 | 0.26 | 0.608 | 1.07(0.83-1.38) | 0.606 | 1.07(0.83-1.38) | 0.597 |
|  | rs2128821 | AA | C | 0.40\0.44 | 1.62 | 0.203 | 0.88(0.72-1.07) | 0.176 | 0.87(0.72-1.06) | 0.190 |
|  |  | EA | G | 0.26\0.24 | 0.17 | 0.681 | 1.05(0.82-1.36) | 0.641 | 1.06(0.82-1.37) | 0.707 |
|  | rs9298496 | AA | C | 0.39\0.37 | 1.19 | 0.276 | 1.12(0.91-1.37) | 0.286 | 1.12(0.91-1.37) | 0.280 |
|  |  | EA | C | 0.33\0.33 | 0.04 | 0.849 | 0.98(0.77-1.24) | 0.936 | 0.98(0.79-1.25) | 0.856 |
|  | rs6981243 | AA | A | 0.4\0.44 | 2.17 | 0.141 | 0.86(0.71-1.05) | 0.147 | 0.86(0.71-1.05) | 0.138 |
|  |  | EA | C | 0.43\0.39 | 1.64 | 0.200 | 1.16(0.93-1.45) | 0.260 | 1.14(0.91-1.42) | 0.202 |
|  | rs7009781 | AA | T | 0.26\0.26 | 0.11 | 0.736 | 1.04(0.83-1.30) | 0.825 | 1.03(0.82-1.28) | 0.728 |
|  |  | EA | C | 0.19\0.17 | 0.74 | 0.390 | 1.13(0.85-1.50) | 0.357 | 1.14(0.86-1.51) | 0.389 |

RA, reference allele; Frequency, RA frequency in case/control; *P_obs_*, observed *P* values using the Pearson’s Chi-square test; *P_adj_*, adjusted *P* values calculated by multivariate logistic regression after adjustment by sex, age and ancestry proportion, under the additive model; *P_emp_*, empirical *P* values calculated from 10,000 times of permutations test; OR, odds ratio; 95%CI, 95% confidence interval.

**Table S3 Haplotype-based association analysis of *RGS17* SNPs in African Americans**

| **Block** | **Haplotype** | **Phenotype** | **Frequencies** | ***Х^2^*** | ***P_obs_*** | ***P_emp_*** |
| --- | --- | --- | --- | --- | --- | --- |
| Block I | ATCCT | AD | 0.274\ 0.256 | 0.850 | 0.357 | 0.999 |
|  |  | CD | 0.266\ 0.256 | 0.256 | 0.613 | 1.000 |
|  |  | OD | 0.271\ 0.256 | 0.425 | 0.515 | 1.000 |
|  |  | MjD | 0.256\ 0.256 | 0.000 | 0.993 | 1.000 |
|  | GATCT | AD | 0.022\ 0.026 | 0.359 | 0.549 | 1.000 |
|  |  | CD | 0.020\ 0.026 | 0.760 | 0.383 | 1.000 |
|  |  | OD | 0.024\ 0.026 | 0.067 | 0.796 | 1.000 |
|  |  | MjD | 0.024\ 0.026 | 0.056 | 0.813 | 1.000 |
|  | GATTC | AD | 0.053\ 0.032 | 5.094 | **0.024** | 0.319 |
|  |  | CD | 0.053\ 0.032 | 5.410 | **0.020** | 0.267 |
|  |  | OD | 0.067\ 0.032 | 9.246 | **0.002** | **0.026** |
|  |  | MjD | 0.057\ 0.032 | 5.950 | **0.015** | 0.178 |
|  | GATTT | AD | 0.222\ 0.232 | 0.290 | 0.590 | 1.000 |
|  |  | CD | 0.222\ 0.232 | 0.307 | 0.580 | 1.000 |
|  |  | OD | 0.230\ 0.232 | 0.011 | 0.915 | 1.000 |
|  |  | MjD | 0.235\ 0.232 | 0.014 | 0.905 | 1.000 |
|  | GTCCT | AD | 0.119\ 0.107 | 0.617 | 0.432 | 1.000 |
|  |  | CD | 0.119\ 0.107 | 0.672 | 0.413 | 1.000 |
|  |  | OD | 0.111\ 0.107 | 0.053 | 0.817 | 1.000 |
|  |  | MjD | 0.113\ 0.107 | 0.107 | 0.743 | 1.000 |
|  | GTCTT | AD | 0.075\ 0.084 | 0.655 | 0.418 | 1.000 |
|  |  | CD | 0.076\ 0.084 | 0.533 | 0.465 | 1.000 |
|  |  | OD | 0.066\ 0.084 | 1.637 | 0.201 | 0.979 |
|  |  | MjD | 0.072\ 0.084 | 0.844 | 0.358 | 1.000 |
|  | GTTCT | AD | 0.015\ 0.029 | 5.833 | **0.016** | 0.222 |
|  |  | CD | 0.016\ 0.029 | 5.441 | **0.020** | 0.265 |
|  |  | OD | 0.010\ 0.029 | 6.650 | **0.010** | 0.106 |
|  |  | MjD | 0.010\ 0.029 | 8.435 | **0.004** | **0.048** |
|  | GTTTT | AD | 0.213\ 0.224 | 0.413 | 0.520 | 1.000 |
|  |  | CD | 0.221\ 0.224 | 0.031 | 0.861 | 1.000 |
|  |  | OD | 0.215\ 0.224 | 0.172 | 0.678 | 1.000 |
|  |  | MjD | 0.228\ 0.224 | 0.031 | 0.859 | 1.000 |
| Block II | CCC | AD | 0.478\ 0.453 | 1.272 | 0.259 | 0.990 |
|  |  | CD | 0.472\ 0.453 | 0.768 | 0.381 | 1.000 |
|  |  | OD | 0.470\ 0.453 | 0.393 | 0.531 | 1.000 |
|  |  | MjD | 0.477\ 0.453 | 1.008 | 0.315 | 0.999 |
|  | CCT | AD | 0.044\ 0.032 | 1.938 | 0.164 | 0.941 |
|  |  | CD | 0.039\ 0.032 | 0.696 | 0.404 | 1.000 |
|  |  | OD | 0.056\ 0.032 | 4.492 | **0.034** | 0.375 |
|  |  | MjD | 0.051\ 0.032 | 3.438 | 0.064 | 0.621 |
|  | CGT | AD | 0.015\ 0.009 | 1.486 | 0.223 | 0.981 |
|  |  | CD | 0.016\ 0.009 | 1.644 | 0.200 | 0.972 |
|  |  | OD | 0.017\ 0.009 | 1.795 | 0.180 | 0.963 |
|  |  | MjD | 0.015\ 0.009 | 1.260 | 0.262 | 0.991 |
|  | TGC | AD | 0.013\ 0.011 | 0.072 | 0.789 | 1.000 |
|  |  | CD | 0.015\ 0.011 | 0.509 | 0.476 | 1.000 |
|  |  | OD | 0.017\ 0.011 | 0.908 | 0.341 | 1.000 |
|  |  | MjD | 0.015\ 0.011 | 0.405 | 0.525 | 1.000 |
|  | TGT | AD | 0.449\ 0.491 | 3.691 | 0.055 | 0.574 |
|  |  | CD | 0.457\ 0.491 | 2.587 | 0.108 | 0.838 |
|  |  | OD | 0.437\ 0.491 | 4.156 | **0.042** | 0.436 |
|  |  | MjD | 0.440\ 0.491 | 4.350 | **0.037** | 0.405 |
| Block III | CC | AD | 0.509\ 0.472 | 2.832 | 0.092 | 0.772 |
|  |  | CD | 0.513\ 0.472 | 3.665 | 0.056 | 0.579 |
|  |  | OD | 0.493\ 0.472 | 0.614 | 0.433 | 1.000 |
|  |  | MjD | 0.528\ 0.472 | 5.354 | **0.021** | 0.240 |
|  | GC | AD | 0.254\ 0.270 | 0.711 | 0.399 | 1.000 |
|  |  | CD | 0.247\ 0.270 | 1.544 | 0.214 | 0.979 |
|  |  | OD | 0.265\ 0.270 | 0.036 | 0.850 | 1.000 |
|  |  | MjD | 0.243\ 0.270 | 1.515 | 0.218 | 0.979 |
|  | GG | AD | 0.238\ 0.259 | 1.215 | 0.270 | 0.992 |
|  |  | CD | 0.241\ 0.259 | 0.947 | 0.331 | 1.000 |
|  |  | OD | 0.242\ 0.259 | 0.505 | 0.478 | 1.000 |
|  |  | MjD | 0.228\ 0.259 | 2.114 | 0.146 | 0.936 |

Frequency, haplotype frequency in case/control; *P_obs_*, observed *P* values using the Pearson’s Chi-square test; *P_emp_*, empirical *P* values calculated from 10,000 times of permutations tests.

**Table S4 Haplotype-based association analysis of *RGS17* SNPs in European Americans**

| **Block** | **Haplotype** | **code** | **Frequencies** | ***Х^2^*** | ***P_obs_*** | ***P_emp_*** |
| --- | --- | --- | --- | --- | --- | --- |
| Block I | ATCCT | AD | 0.252\ 0.247 | 0.059 | 0.808 | 1.000 |
|  |  | CD | 0.243\ 0.247 | 0.034 | 0.853 | 1.000 |
|  |  | OD | 0.252\ 0.247 | 0.058 | 0.810 | 1.000 |
|  |  | MjD | 0.246\ 0.247 | 0.002 | 0.963 | 1.000 |
|  | GATTC | AD | 0.322\ 0.318 | 0.029 | 0.864 | 1.000 |
|  |  | CD | 0.333\ 0.318 | 0.498 | 0.480 | 0.999 |
|  |  | OD | 0.328\ 0.318 | 0.214 | 0.643 | 1.000 |
|  |  | MjD | 0.316\ 0.318 | 0.003 | 0.958 | 1.000 |
|  | GTCTT | AD | 0.211\ 0.258 | 5.745 | **0.017** | 0.159 |
|  |  | CD | 0.206\ 0.258 | 7.136 | **0.008** | 0.070 |
|  |  | OD | 0.210\ 0.258 | 5.555 | **0.018** | 0.169 |
|  |  | MjD | 0.212\ 0.258 | 3.820 | 0.051 | 0.382 |
|  | GTTCT | AD | 0.047\ 0.037 | 1.251 | 0.263 | 0.991 |
|  |  | CD | 0.053\ 0.037 | 2.743 | 0.098 | 0.709 |
|  |  | OD | 0.053\ 0.037 | 2.557 | 0.110 | 0.764 |
|  |  | MjD | 0.052\ 0.037 | 1.888 | 0.169 | 0.904 |
|  | GTTTT | AD | 0.138\ 0.119 | 1.478 | 0.224 | 0.965 |
|  |  | CD | 0.136\ 0.119 | 1.186 | 0.276 | 0.984 |
|  |  | OD | 0.129\ 0.119 | 0.456 | 0.499 | 1.000 |
|  |  | MjD | 0.150\ 0.119 | 2.835 | 0.092 | 0.643 |
| Block II | CCCC | AD | 0.295\ 0.260 | 2.810 | 0.094 | 0.682 |
|  |  | CD | 0.302\ 0.260 | 4.073 | **0.044** | 0.387 |
|  |  | OD | 0.324\ 0.260 | 8.256 | **0.004** | **0.028** |
|  |  | MjD | 0.309\ 0.260 | 3.981 | **0.046** | 0.345 |
|  | TGCC | AD | 0.068\ 0.081 | 1.092 | 0.296 | 0.996 |
|  |  | CD | 0.065\ 0.081 | 1.603 | 0.206 | 0.946 |
|  |  | OD | 0.070\ 0.081 | 0.693 | 0.405 | 0.999 |
|  |  | MjD | 0.082\ 0.081 | 0.007 | 0.933 | 1.000 |
|  | TGTC | AD | 0.072\ 0.087 | 1.558 | 0.212 | 0.955 |
|  |  | CD | 0.067\ 0.087 | 2.879 | 0.090 | 0.662 |
|  |  | OD | 0.071\ 0.087 | 1.656 | 0.198 | 0.955 |
|  |  | MjD | 0.077\ 0.087 | 0.485 | 0.486 | 1.000 |
|  | TGTT | AD | 0.550\ 0.558 | 0.135 | 0.713 | 1.000 |
|  |  | CD | 0.551\ 0.558 | 0.111 | 0.739 | 1.000 |
|  |  | OD | 0.525\ 0.558 | 1.928 | 0.165 | 0.915 |
|  |  | MjD | 0.511\ 0.558 | 3.029 | 0.082 | 0.610 |
| Block III | CC | AD | 0.432\ 0.420 | 0.250 | 0.617 | 1.000 |
|  |  | CD | 0.445\ 0.420 | 1.160 | 0.282 | 0.985 |
|  |  | OD | 0.471\ 0.420 | 4.558 | 0.033 | 0.317 |
|  |  | MjD | 0.447\ 0.420 | 0.965 | 0.326 | 0.997 |
|  | GC | AD | 0.107\ 0.112 | 0.107 | 0.743 | 1.000 |
|  |  | CD | 0.098\ 0.112 | 0.980 | 0.322 | 0.995 |
|  |  | OD | 0.099\ 0.112 | 0.787 | 0.375 | 0.998 |
|  |  | MjD | 0.112\ 0.112 | 0.000 | 0.990 | 1.000 |
|  | GG | AD | 0.455\ 0.465 | 0.208 | 0.649 | 1.000 |
|  |  | CD | 0.454\ 0.465 | 0.249 | 0.618 | 1.000 |
|  |  | OD | 0.424\ 0.465 | 2.964 | 0.085 | 0.678 |
|  |  | MjD | 0.439\ 0.465 | 0.948 | 0.330 | 0.997 |

Frequency, haplotype frequency in case/control; *P_obs_*, observed *P* values using the Pearson’s Chi-square test; *P_emp_*, empirical *P* values calculated from 10,000 times of permutations tests.

| **Table S5 Association of genotypes of *RGS17* SNPs with *RGS17* expression levels** | | | | | | | | | |  |
| --- | --- | --- | --- | --- | --- | --- | --- | --- | --- | --- |
| **RGS17** |  | ***RGS17* expression in CEU** | | | |  | ***RGS17* expression in YRI** | | | |
| **SNPs** | **Genotypes** | **Mean** | **S.D** | **T** | ***P*** |  | **Mean** | **S.D** | **T** | ***P*** |
| rs9397578 | AA | 6.67 | 0.50 |  |  |  | 6.51 | 0.40 |  |  |
|  | AG | 6.84 | 0.66 | 1.78 | 0.080 |  | 6.57 | 0.44 | 0.45 | 0.655 |
|  | GG | 7.38 | 1.19 |  |  |  | 6.59 | 0.35 |  |  |
| rs7750874 | TT | 6.83 | 0.72 |  |  |  | 6.48 | 0.38 |  |  |
|  | AT | 6.65 | 0.50 | -0.69 | 0.491 |  | 6.65 | 0.45 | 0.53 | 0.596 |
|  | AA | 6.83 | 0.56 |  |  |  | 6.42 | 0.28 |  |  |
| rs503366 | TT | 6.49 | 0.41 |  |  |  | 6.48 | 0.35 |  |  |
|  | CT | 6.81 | 0.58 | 2.24 | **0.029** |  | 6.63 | 0.50 | -0.31 | 0.758 |
|  | CC | 7.01 | 0.79 |  |  |  | 6.43 | 0.24 |  |  |
| rs610614 | TT | 6.71 | 0.54 |  |  |  | 6.65 | 0.46 |  |  |
|  | CT | 6.74 | 0.62 | 0.82 | 0.413 |  | 6.52 | 0.42 | -0.94 | 0.353 |
|  | CC | 7.05 | 1.17 |  |  |  | 6.50 | 0.27 |  |  |
| rs545323 | TT | 6.90 | 0.72 |  |  |  | 6.54 | 0.40 |  |  |
|  | CT | 6.66 | 0.51 | -0.97 | 0.337 |  | N/A | N/A | N/A | N/A |
|  | CC | 6.83 | 0.56 |  |  |  | N/A | N/A |  |  |
| rs516557 | TT | 6.77 | 0.55 |  |  |  | 6.49 | 0.23 |  |  |
|  | CT | 6.74 | 0.66 | -0.17 | 0.864 |  | 6.48 | 0.41 | 1.14 | 0.259 |
|  | CC | 6.74 | 0.64 |  |  |  | 6.64 | 0.47 |  |  |
| rs9371276 | TT | 6.90 | 0.73 |  |  |  | 6.70 | 0.47 |  |  |
|  | CT | 6.57 | 0.42 | -1.65 | 0.105 |  | 6.55 | 0.39 | -2.32 | **0.024** |
|  | CC | 6.63 | 0.45 |  |  |  | 6.35 | 0.29 |  |  |
| rs9397585 | TT | 7.05 | 0.73 |  |  |  | 6.68 | 0.43 |  |  |
|  | CT | 6.60 | 0.45 | -2.73 | **0.009** |  | 6.52 | 0.42 | -2.05 | **0.045** |
|  | CC | 6.54 | 0.43 |  |  |  | 6.37 | 0.28 |  |  |
| rs685826 | TT | 6.80 | 0.71 |  |  |  | 6.70 | 0.57 |  |  |
|  | CT | 6.77 | 0.59 | -0.60 | 0.554 |  | 6.60 | 0.37 | 2.64 | **0.011** |
|  | CC | 6.67 | 0.55 |  |  |  | 6.29 | 0.21 |  |  |
| rs6931160 | CC | 6.53 | 0.43 |  |  |  | 6.29 | 0.24 |  |  |
|  | CG | 6.65 | 0.46 | 2.40 | **0.020** |  | 6.54 | 0.39 | 3.13 | **0.003** |
|  | GG | 7.02 | 0.81 |  |  |  | 6.74 | 0.42 |  |  |
| rs1281962 | CC | 6.61 | 0.52 |  |  |  | 6.42 | 0.31 |  |  |
|  | CG | 6.77 | 0.57 | -1.69 | 0.097 |  | 6.65 | 0.45 | 2.50 | **0.015** |
|  | GG | 6.98 | 0.85 |  |  |  | 6.95 | N/A |  |  |
| rs596359 | AA | 6.93 | 0.74 |  |  |  | 6.69 | 0.46 |  |  |
|  | AG | 6.74 | 0.58 | -1.34 | 0.185 |  | 6.40 | 0.27 | -3.25 | **0.002** |
|  | GG | 6.61 | 0.47 |  |  |  | 6.23 | 0.06 |  |  |

The association of *RGS17* SNP genotypes and *RGS17* expression levels was analyzed using linear regression under additive model adjusted by sex

| **Table S6 Association of genotypes of *RGS17* SNPs with *OPRM1* expression levels** | | | | | | | | | |  |
| --- | --- | --- | --- | --- | --- | --- | --- | --- | --- | --- |
| **RGS17** |  | ***OPRM1* expression in CEU** | | | |  | ***OPRM1* expression in YRI** | | | |
| **SNPs** | **Genotypes** | **Mean** | **S.D.** | **T** | ***P*** |  | **Mean** | **S.D** | **T** | ***P*** |
| rs9397578 | AA | 6.23 | 0.07 |  |  |  | 6.23 | 0.06 |  |  |
|  | AG | 6.25 | 0.08 | 0.72 | 0.475 |  | 6.23 | 0.06 | 0.39 | 0.695 |
|  | GG | 6.22 | 0.04 |  |  |  | 6.24 | 0.10 |  |  |
| rs7750874 | TT | 6.23 | 0.08 |  |  |  | 6.24 | 0.05 |  |  |
|  | AT | 6.23 | 0.08 | 0.17 | 0.865 |  | 6.23 | 0.07 | -1.01 | 0.316 |
|  | AA | 6.25 | 0.03 |  |  |  | 6.21 | 0.05 |  |  |
| rs503366 | TT | 6.25 | 0.08 |  |  |  | 6.23 | 0.06 |  |  |
|  | CT | 6.22 | 0.07 | -0.16 | 0.877 |  | 6.23 | 0.07 | -0.03 | 0.976 |
|  | CC | 6.25 | 0.06 |  |  |  | 6.23 | 0.06 |  |  |
| rs610614 | TT | 6.23 | 0.07 |  |  |  | 6.22 | 0.07 |  |  |
|  | CT | 6.24 | 0.08 | 0.54 | 0.591 |  | 6.24 | 0.05 | 0.58 | 0.596 |
|  | CC | 6.23 | 0.03 |  |  |  | 6.23 | 0.06 |  |  |
| rs545323 | TT | 6.24 | 0.07 |  |  |  | 6.23 | 0.06 |  |  |
|  | CT | 6.23 | 0.08 | -0.06 | 0.950 |  | N/A | N/A |  | N/A |
|  | CC | 6.25 | 0.03 |  |  |  | N/A | N/A |  |  |
| rs516557 | TT | 6.24 | 0.07 |  |  |  | 6.24 | 0.07 |  |  |
|  | CT | 6.22 | 0.08 | 0.06 | 0.952 |  | 6.23 | 0.06 | -0.43 | 0.671 |
|  | CC | 6.25 | 0.07 |  |  |  | 6.23 | 0.06 |  |  |
| rs9371276 | TT | 6.23 | 0.08 |  |  |  | 6.25 | 0.06 |  |  |
|  | CT | 6.22 | 0.08 | 0.21 | 0.831 |  | 6.23 | 0.06 | -0.97 | 0.338 |
|  | CC | 6.25 | 0.05 |  |  |  | 6.22 | 0.05 |  |  |
| rs9397585 | TT | 6.24 | 0.08 |  |  |  | 6.25 | 0.06 |  |  |
|  | CT | 6.22 | 0.08 | 0.60 | 0.552 |  | 6.23 | 0.07 | -1.11 | 0.274 |
|  | CC | 6.26 | 0.05 |  |  |  | 6.22 | 0.06 |  |  |
| rs685826 | TT | 6.24 | 0.09 |  |  |  | 6.23 | 0.06 |  |  |
|  | CT | 6.22 | 0.07 | -0.22 | 0.828 |  | 6.23 | 0.06 | -0.41 | 0.682 |
|  | CC | 6.23 | 0.06 |  |  |  | 6.23 | 0.05 |  |  |
| rs6931160 | CC | 6.27 | 0.05 |  |  |  | 6.20 | 0.06 |  |  |
|  | CG | 6.20 | 0.07 | -0.45 | 0.654 |  | 6.24 | 0.06 | 1.69 | 0.096 |
|  | GG | 6.25 | 0.07 |  |  |  | 6.24 | 0.06 |  |  |
| rs1281962 | CC | 6.25 | 0.06 |  |  |  | 6.22 | 0.06 |  |  |
|  | CG | 6.21 | 0.07 | -0.19 | 0.849 |  | 6.24 | 0.06 | 1.69 | 0.097 |
|  | GG | 6.25 | 0.09 |  |  |  | 6.36 | N/A |  |  |
| rs596359 | AA | 6.25 | 0.06 |  |  |  | 6.24 | 0.06 |  |  |
|  | AG | 6.23 | 0.08 | -1.37 | 0.176 |  | 6.23 | 0.06 | -1.24 | 0.220 |
|  | GG | 6.21 | 0.07 |  |  |  | 6.19 | 0.03 |  |  |

The association of *RGS17* SNP genotypes and *OPRM1* expression levels was analyzed using linear regression under additive model adjusted by sex.

**
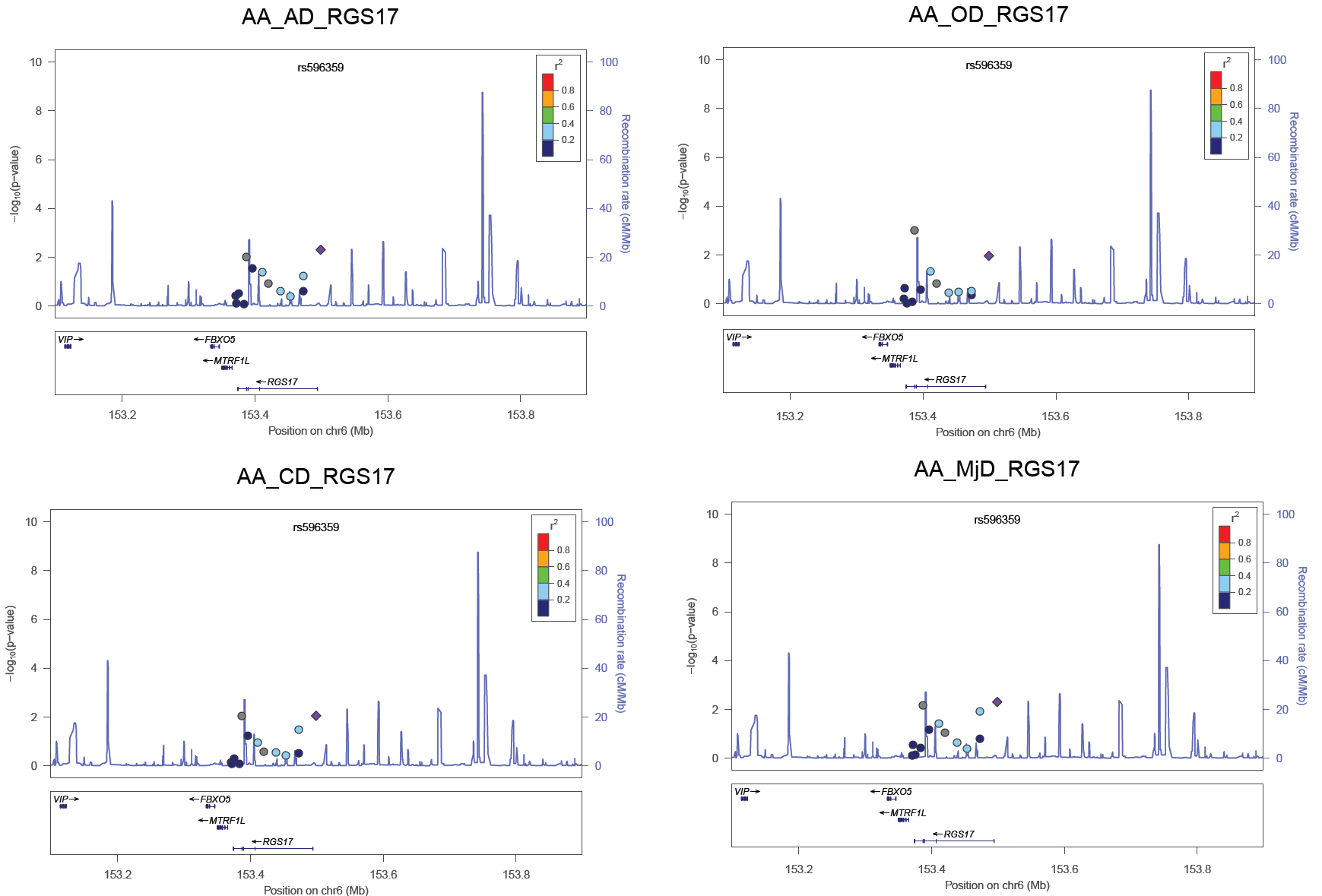
**

**Figure S1 Regional plots of the association between 13 *RGS17* SNPs and substance dependence in African Americans (AAs).**

Regional plots of the association of 13 *RGS17*SNPs and alcohol, cocaine, opioid, or marijuana dependence (AD, CD, OD or MjD) in African Americans (AAs) were generated using program LocusZoom (http://csg.sph.umich.edu/locuszoom). The left Y axis represents the -log_10_(*P* value) (*P* values were obtained from genetic association analyses of 13 *RGS17* SNPs). Each dot represents a SNP marker; color in dot represents the R square value (indicated by color bar on top right corner, from blue to red) between SNPs and reference SNP rs596359 (marked with purple). The right Y axis and the blue curve on the X axis represent the recombination rate in the *RGS17* gene region. Genomic position and annotation within genes are shown under the X axis [these data are obtained from HapMap hg18 (CEU or YRI) dataset].


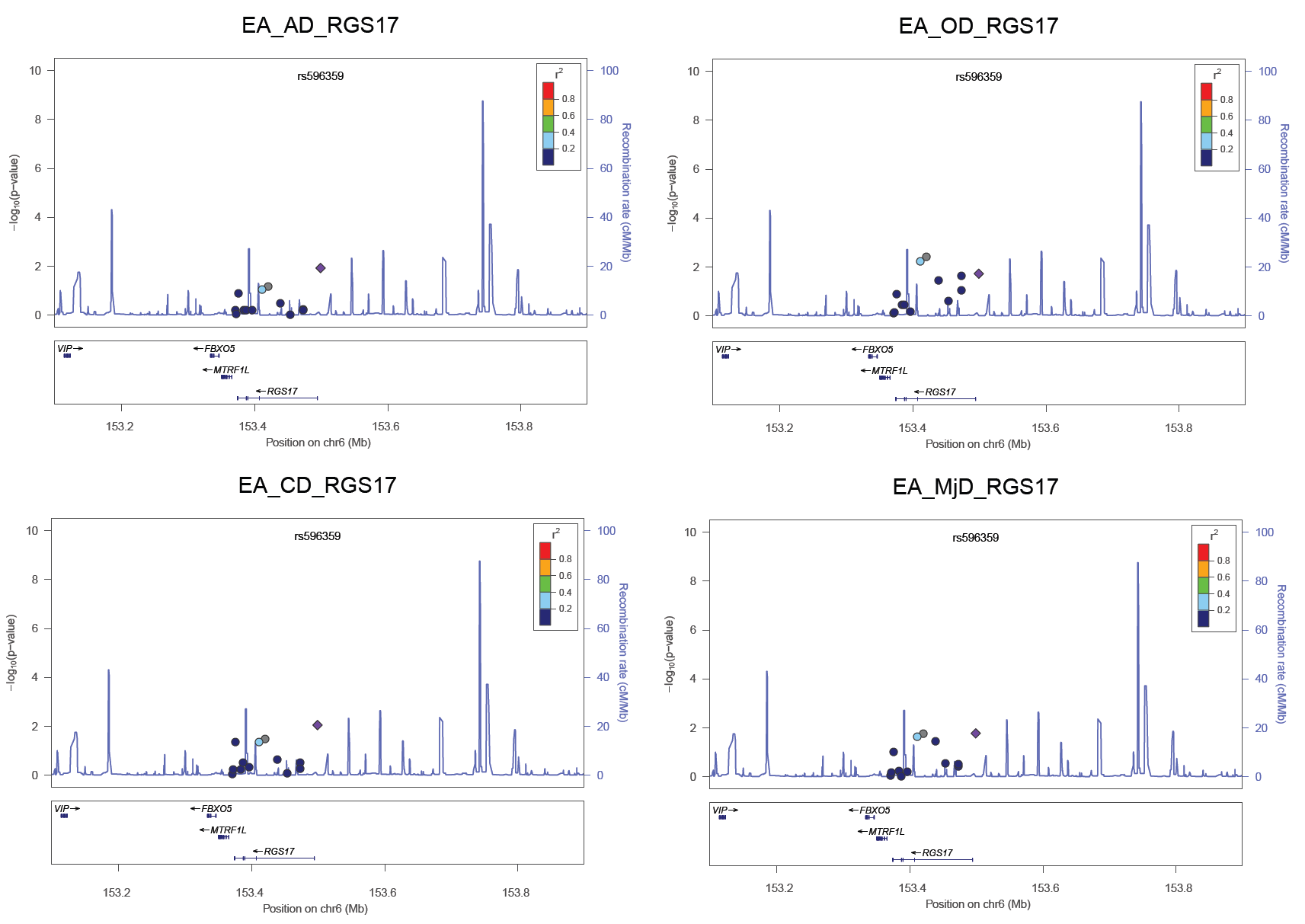


**Figure S2 Regional plots of the association between 13 *RGS17* SNPs and substance dependence in European Americans (EAs).**

Regional plots of the association of 13 *RGS17*SNPs and alcohol, cocaine, opioid, or marijuana dependence (AD, CD, OD or MjD) in European Americans (EAs) were generated using program LocusZoom (http://csg.sph.umich.edu/locuszoom). The left Y axis represents the -log_10_(*P* value) (*P* values were obtained from genetic association analyses of 13 *RGS17* SNPs). Each dot represents a SNP marker; color in dot represents the R square value (indicated by color bar on top right corner, from blue to red) between SNPs and reference SNP rs596359 (marked with purple). The right Y axis and the blue curve on the X axis represent the recombination rate in the *RGS17* gene region. Genomic position and annotation within genes are shown under the X axis [these data are obtained from HapMap hg18 (CEU or YRI) dataset].
